# Curcumin–Piperine Self-Nanoemulsifying Delivery in *Zanthoxylum rhetsa* Seed Oil Attenuates Cuprizone-Induced Frontal Cortex Toxicity

**DOI:** 10.3390/ph18101478

**Published:** 2025-10-01

**Authors:** Mohammad Zubair Alam, Hala Abubaker Bagabir, Mohammad Alameen Faisal Zaher, Thamer M. A. Alqurashi, Badrah S. Alghamdi, Mohsin Kazi, Gamal Said Abd El-Aziz, Gadah Ali Alshahrany, Noor Ahmed Alzahrani, Rafal Mohammed Bakhalgi, Mona Al-Thepyani, Hanin Abdulbaset AboTaleb, Rahaf Saeed Aldhahri, Juweiriya, Ghulam Md Ashraf

**Affiliations:** 1 Neuroscience and Geroscience Research Unit, King Fahd Medical Research Center, King Abdulaziz University, Jeddah 21589, Saudi Arabia; mzalam@kau.edu.sa (M.Z.A.); basalghamdi@kau.edu.sa (B.S.A.); 2 Department of Medical Laboratory Technology, Faculty of Applied Medical Sciences, King Abdulaziz University, Jeddah 21589, Saudi Arabia; 3 Department of Physiology, Faculty of Medicine, King Abdulaziz University, Rabigh 25732, Saudi Arabia; 4 Department of Physiology, Faculty of Medicine, King Abdulaziz University, Jeddah 21589, Saudi Arabia; 5 Department of Pharmacology, Faculty of Medicine, King Abdulaziz University, Rabigh 25732, Saudi Arabia; 6 Neuroscience Unit, Department of Physiology, Faculty of Medicine, King Abdulaziz University, Jeddah 21589, Saudi Arabia; 7 Department of Pharmaceutics, College of Pharmacy, King Saud University, P.O. Box 2457, Riyadh 11451, Saudi Arabia; mkazi@ksu.edu.sa; 8 Department of Clinical Anatomy, Faculty of Medicine, King Abdulaziz University, Jeddah 21589, Saudi Arabia; dr_gamal_said@yahoo.com; 9 Department of Biochemistry, Faculty of Sciences, King Abdulaziz University, Jeddah 21589, Saudi Arabia; 10 Department of Microbiology, Faculty of Science, King Abdulaziz University, Jeddah 21589, Saudi Arabia; 11 Department of Chemistry, College of Sciences & Arts, King Abdulaziz University, Rabigh 25732, Saudi Arabia; 12 Department of Biological Sciences, Faculty of Sciences, University of Jeddah, Djedda 21589, Saudi Arabia; 13 Department of Chemistry, Aligarh Muslim University, Aligarh 202002, India; 14 Department of Biomedical Sciences, College of Medicine, Gulf Medical University, Ajman P.O. Box 4184, United Arab Emirates

**Keywords:** multiple sclerosis, demyelination, neuroinflammation, cuprizone, frontal cortex

## Abstract

**Background/Objectives:** Demyelination and neuroinflammation are central features of multiple sclerosis (MS), contributing to motor deficits and cognitive decline. Cuprizone (CPZ)-induced demyelination is a well-established model for studying multiple sclerosis-like neurotoxicity. This study investigated the neuroprotective and immunomodulatory effects of self-nanoemulsifying drug delivery systems (SNEDDSs) incorporating curcumin, piperine, and *Zanthoxylum rhetsa* seed oil. **Methods:** Male mice were divided into five groups: control, CPZ-only, and CPZ co-treated with three nanoformulations BFZ (blank SNEDDS), CFZ (curcumin-SNEDDS), and PFZ (curcumin–piperine SNEDDS). CPZ was administered for 5 weeks, followed by a 2-week recovery or treatment phase. Key neuroinflammatory markers like CD4, CD8, cholinergic (acetylcholinesterase, AChE), myelin integrity (MBP), BDNF, CREB, TNFα, Il-1β were assessed at weeks 5 and 7 using ELISA. Alterations in antioxidant enzymes, brain histology, and behavioral outcomes were also investigated. **Results:** At week 5, CPZ significantly increased CD4 and CD8 expression and reduced AChE and MBP levels, indicating neuroinflammation, cholinergic impairment, and demyelination. Nanoformulation treatments (both prophylactic and therapeutic) markedly reduced CD4 and CD8 levels, with PFZ showing the most pronounced effect. AChE activity was significantly restored in all treatment groups, with PFZ and CFZ exceeding baseline levels, suggesting enhanced cholinergic function. MBP levels were highest in PFZ-treated mice, surpassing control values and indicating strong remyelination potential. These improvements persisted and further advanced at week 7, especially in PFZ and CFZ groups. **Conclusions:** Curcumin-based SNEDDS, particularly PFZ, significantly mitigated CPZ-induced neuroinflammation, promoted remyelination, and restored cholinergic activity in the frontal cortex. These findings highlight the therapeutic potential of bioenhanced curcumin nanoformulations for treating demyelinating and neuroinflammatory disorders.

## 1. Introduction

In 1868, Jean-Martin Charcot a French neurologist provided the first detailed clinical and pathological characterization of MS, identifying hallmark features such as demyelination and perivascular inflammation in the central nervous system [1]. His observations laid the groundwork for understanding MS as a distinct neurological disorder. In the early 20th century, researchers began developing experimental models to study MS, most notably the experimental autoimmune encephalomyelitis (EAE) model, which mimicked immune-mediated demyelination and became instrumental in studying MS pathogenesis and testing therapeutic interventions [2]. These pioneering efforts established the foundation for current MS research, including both immunological and neurodegenerative components of the disease. Multiple sclerosis (MS) is a chronic, immune-mediated neurodegenerative disorder of the central nervous system (CNS), characterized by demyelination, axonal injury, and neuroinflammation [3]. Affecting millions of individuals globally, MS exhibits a complex etiology involving genetic predisposition, environmental triggers, and autoimmune dysregulation. Hallmark pathological features of MS include demyelination, astrogliosis, microglial activation, and subsequent neuronal loss, which collectively contribute to the progressive neurological decline observed in patients [4]. Despite significant advances in MS research, understanding its underlying mechanisms remains challenging, necessitating the use of reliable animal models to replicate aspects of human disease for preclinical investigations.

One of the extensively used models for studying MS pathophysiology is the cuprizone (CPZ) mouse model. CPZ, a copper-chelating agent, when administered to mice through their diet, induces robust demyelination in specific brain regions, particularly the corpus callosum, hippocampus, and cortex [5]. Unlike autoimmune models such as experimental autoimmune encephalomyelitis (EAE), CPZ-induced demyelination is primarily mediated through oligodendrocyte apoptosis and microglial activation without direct peripheral immune cell infiltration [6]. Therefore, the CPZ model is particularly valuable for studying CNS intrinsic mechanisms of demyelination and remyelination in the absence of an overt peripheral immune response.

Behavioral assessments in CPZ-treated mice are critical for evaluating the functional consequences of demyelination and therapeutic interventions. One of the commonly used parameters is total distance moved and velocity, typically measured using open-field tests. These metrics provide insight into the general locomotor activity, exploratory behavior, and motor coordination, which are often impaired in demyelinating conditions. Reduced locomotor activity and velocity are indicative of underlying neurodegenerative changes, highlighting the relevance of behavioral testing alongside histological and molecular assessments. Another vital behavioral assay is the grip strength test, which measures forelimb and/or hindlimb muscle strength in rodents. Grip strength serves as an indicator of neuromuscular integrity and is sensitive to CNS demyelination and axonal dysfunction [7]. CPZ-induced demyelination has been associated with significant reductions in grip strength, correlating with motor deficits observed in MS patients. Therefore, behavioral phenotyping through these tests provides a functional correlate to the histopathological changes occurring in the brain.

Oxidative stress is increasingly recognized as a pivotal contributor to MS pathology. Reactive oxygen species (ROS) generated during neuroinflammation can exacerbate demyelination and neuronal injury [8]. The CNS possesses an intrinsic antioxidant defense system composed of enzymatic and non-enzymatic molecules. Among these, superoxide dismutase (SOD), catalase (CAT), glutathione peroxidase (GPX), and reduced glutathione (GSH) are essential for mitigating oxidative damage. SOD catalyzes the dismutation of superoxide radicals into hydrogen peroxide, which is subsequently decomposed into water and oxygen by catalase [9]. GPX, in conjunction with GSH, reduces lipid hydroperoxides and hydrogen peroxide, thus protecting cellular membranes and proteins from oxidative insults [10]. A decline in the activities of these antioxidant enzymes has been documented in MS lesions and correlates with disease severity [11].

In the CPZ model, oxidative stress is an early and sustained event that precedes and accompanies demyelination. Therefore, quantifying the levels and activities of these antioxidant molecules offers valuable insights into the oxidative mechanisms underlying CPZ-induced neurotoxicity and the potential neuroprotective effects of therapeutic agents. Neuroinflammation is a hallmark of MS and is closely intertwined with oxidative stress. Key inflammatory markers implicated in MS include pro-inflammatory cytokines such as tumor necrosis factor-alpha (TNF-α) and interleukin-1 beta (IL-1β). Elevated levels of TNF-α and IL-1β have been detected in MS lesions and cerebrospinal fluid of patients, contributing to oligodendrocyte apoptosis, blood–brain barrier disruption, and demyelination [12]. In CPZ-treated mice, similar upregulation of TNF-α and IL-1β has been observed, reinforcing the model’s relevance to MS-associated neuroinflammation [13].

In addition to classical pro-inflammatory cytokines, neurotrophic and transcriptional regulators such as brain-derived neurotrophic factor (BDNF) and cyclic AMP response element-binding protein (CREB) play crucial roles in CNS repair and neuroprotection. BDNF promotes neuronal survival, differentiation, and remyelination, while CREB regulates the expression of several genes involved in neuronal plasticity and survival [14]. Dysregulation of BDNF and CREB signaling has been implicated in MS pathogenesis, with reduced BDNF levels correlating with greater disease severity [15]. In CPZ models, interventions that enhance BDNF and CREB activation have been associated with improved remyelination and functional recovery [16].

Histological evaluation remains a cornerstone for assessing demyelination and remyelination processes in animal models. Luxol fast blue (LFB) staining is a classical histological method used to visualize myelin integrity. LFB selectively stains the myelin sheath, allowing for the quantification of demyelinated areas [17]. In the CPZ model, LFB staining reveals prominent demyelination in the corpus callosum and other white matter tracts, providing a robust and quantifiable measure of myelin loss. Furthermore, combining LFB staining with immunohistochemistry for markers such as myelin basic protein (MBP) or oligodendrocyte lineage markers can yield comprehensive insights into the extent of demyelination, gliosis, and regenerative responses. Hence, the CPZ mouse model serves as a valuable platform to study the mechanisms underlying demyelination, oxidative stress, and neuroinflammation in MS. Behavioral assessments such as total distance moved, velocity, and grip strength provide functional correlates to the pathological alterations. Investigating antioxidant enzyme activities, inflammatory cytokine profiles, and histological changes via LFB staining enables a multi-faceted understanding of CPZ-induced CNS damage. These integrated approaches are crucial for developing and evaluating novel therapeutic strategies aimed at mitigating demyelination and promoting neurodegenerations in MS.

*Zanthoxylum rhetsa* is a medicinally valuable plant belonging to the Rutaceae family. Traditionally used across various Asian medicinal systems, *Z. rhetsa* has been acclaimed for its anti-inflammatory, analgesic, antimicrobial, and antioxidant properties [18]. Various parts of the plant, including its bark, leaves, fruits, and seeds, have been explored for therapeutic applications. Particularly, the seed oil extracted from *Z. rhetsa* has attracted attention due to its rich phytochemical profile. The seed oil of *Zanthoxylum rhetsa* contains a variety of bioactive compounds such as monoterpenes, sesquiterpenes, fatty acids, and alkaloids, contributing to its potent pharmacological activities. Major components identified include limonene, linalool, β-caryophyllene, and other oxygenated terpenes, which confer strong anti-inflammatory and antioxidant effects [19]. Additionally, the oil has demonstrated significant free radical scavenging activity, suggesting its potential in mitigating oxidative stress-related pathological conditions, including neurodegenerative diseases such as MS. The chemical composition of the seed essential oil of *Z. rhetsa* exhibits considerable geographic and environmental variability. In specimens grown in Kerala, South India, the seed essential oil was found to be rich in monoterpenes, with major constituents including sabinene, α-pinene, α-terpinene, β-pinene, γ-terpinene, myrcene, terpinolene, and limonene [20]. In contrast, the seed essential oil from *Z. rhetsa* collected in Northeast India was predominantly composed of terpinen-4-ol (32.1%), followed by α-terpineol (8.2%), sabinene (8.1%), β-phellandrene (7.4%), and 2-undecanone (7.1%) [21]. In an earlier study, characterization of seed oil using Gas Chromatography with Flame Ionization Detection and Gas Chromatography-Mass Spectrometry was conducted. The researchers of the study reported the presence of monoterpene hydrocarbons sabinene, limonene, α-pinene, α-pinene, paracymene and terpinenes, and monoterpene alcohols terpinen-4-ol and α-terpineol, germarene dominantly which altogether give the characteristic aroma of the seed [22].

**Figure 1:**
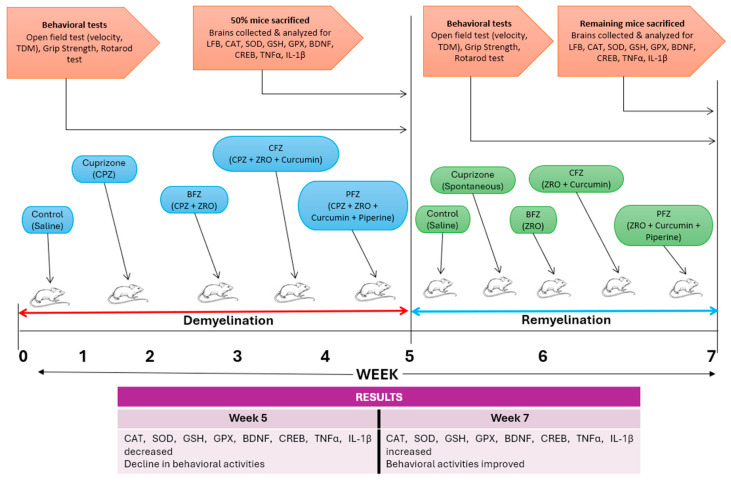
The time scale and graphical representation of the study.

In recent years, nanoformulation strategies have been increasingly utilized to enhance the therapeutic efficacy of natural compounds with poor bioavailability, such as curcumin and piperine. Biomolecular investigations have revealed that curcumin is a pleiotropic molecule capable of modulating numerous signaling molecules and cellular pathways. Despite its low bioavailability, its structural plasticity and wide range of molecular interactions support its potential in the treatment of chronic inflammatory, neurodegenerative, and oncological disorders. Continued research into novel delivery systems and synthetic analogs aims to overcome current limitations and fully harness curcumin’s therapeutic potential [23]. Curcumin suppresses NF-κB activation, thereby reducing the expression of pro-inflammatory cytokines such as TNF-α, IL-1β, and IL-6 [24]. It downregulates p38 and JNK pathways, which are involved in cellular stress and inflammation [25]. Curcumin inhibits PI3K/Akt/mTOR Pathway in cancer cells, contributing to apoptosis and autophagy [26]. Curcumin scavenges reactive oxygen species and reactive nitrogen species (RNS), while also upregulate endogenous antioxidant enzymes such as superoxide dismutase, catalase, and glutathione peroxidase [27]. These effects contribute to its protective role in neurodegenerative diseases and cardiovascular conditions. Piperine, an alkaloid from *Piper nigrum* (black pepper), enhances the bioavailability of curcumin significantly by inhibiting its hepatic and intestinal glucuronidation [28]. To overcome these limitations, nanoemulsions and nanoparticle systems using *Z. rhetsa* seed oil as a carrier have been explored. In such formulations, the seed oil serves not only as a biocompatible lipid matrix but also synergizes with curcumin and piperine due to its intrinsic anti-inflammatory and antioxidant actions. The present investigation evaluated the deleterious impacts of cuprizone on the frontal cortex and the safeguarding effects of curcumins and piperine nanoformulations made in *Zanthoxylum rhetsa* seed oil. These evaluations included modifications to antioxidant enzymes/proteins, transcription factors, and cytokines, in addition to behavioral and histological alterations during the demyelination and remyelination processes. The time scale and graphical representation of the study is presented in Figure 1. *Zanthoxylum rhetsa* seed oil (ZRO) was selected to prepare nanoformulations of curcumin/piperine due to its homogeneity, spontaneity and appearance upon aqueous dispersion. We screened 4 different oils such as black seed oil, apricot oil, avocado oil, *Z. rhetsa* seed oil with various combinations of surfactants and co-surfactants to obtain the most stable emulsion of curcumin and piperine. *Z. rhetsa* seed oil showed the highest magnitude of zeta potential values which gives an indication of system stability. Moreover, it showed highest piperine solubility as well as fairly good curcumin solubility [29].

## 2. Results

### 2.1. GC-MS Analysis of Zanthoxylum rhetsa Seed Oil

**Figure 2:**
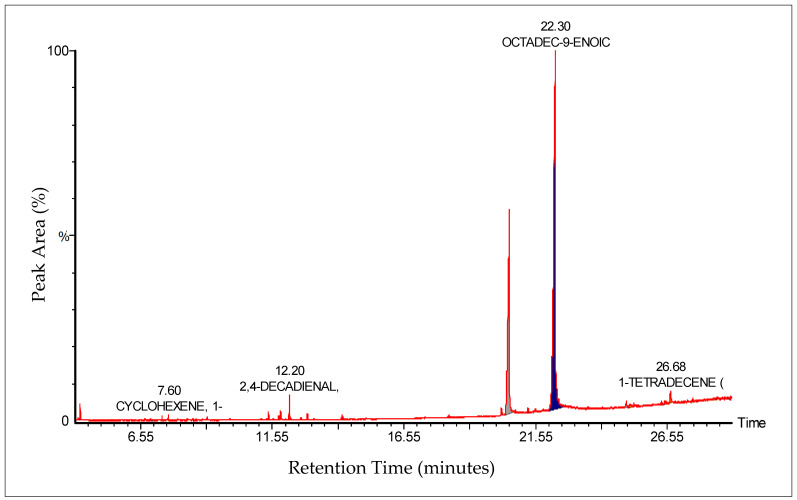
GC-MS chromatogram of *Zanthoxylum rhetsa* seed oil showing major peaks identified.

The GC-MS analysis of *Zanthoxylum rhetsa* seed oil identified 18 compounds spanning fatty acids, aldehydes, alcohols, hydrocarbons, phenolics, sulfur compounds, and lactones, with retention times between 4.24 and 26.69 min. The oil was primarily composed of octadec-9-enoic acid (oleic acid, covering peak area of 59.22%) and hexadecanoic acid (palmitic acid, covering peak area of 28.42%), together contributing nearly 88% of the total peak area. Other notable constituents included 2,4-decadienal, hexanal and 1-ethyl-cyclohexene covering peak area of 4.46%, 1.25%, 1.17%, respectively (Table 1 and Figure 2). The chemical fingerprint was thus fatty acid dominated, with long-chain fatty acids (both unsaturated and saturated) as the principal components, accompanied by a smaller fraction of volatile aldehydes and minor bioactive compounds.

### 2.2. Effects of Cuprizone and Nanoformulations on TDM

**Figure 3:**
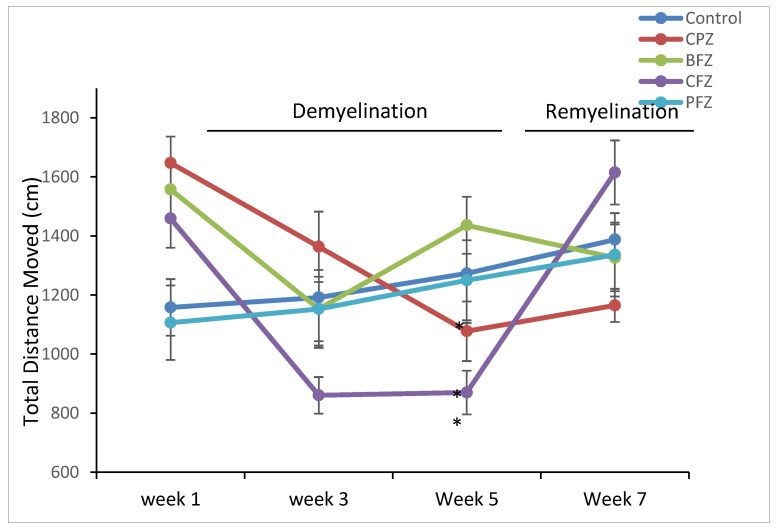
Total distance moved (TDM) as determined in Open Field Test. At week 5 were found significantly reduced in CPZ and CFZ groups compared to the control group. However, TDM appeared restored at the end pf week 7. One-way ANOVA with Tukey’s multiple comparison test was performed to compare the variance between groups. * *p* ≤ 0.05.

Following five weeks of cuprizone (CPZ) administration, the CPZ group exhibited a significant decline in motor activity compared to the control group (*p* ≤ 0.05), as measured by total distance moved (TDM). Specifically, the TDM in the CPZ group (1077.29 ± 83.47 cm) was markedly lower than that of the control (1272.91 ± 168.04 cm) group (Figure 3). This finding confirms the well-documented neurotoxic effects of cuprizone, particularly its ability to impair locomotor function through demyelination and neuroinflammatory mechanisms. Interestingly, mice treated with the blank nanoformulation (BFZ) demonstrated a higher TDM (1436.13 ± 96.31 cm) compared to the CPZ group, even surpassing the control group values, suggesting that the vehicle formulation itself might offer some neuroprotective or supportive effects, potentially due to the antioxidant properties of the seed oil components. Similarly, mice receiving the curcumin–piperine nanoformulation (PFZ) maintained a TDM (1249.77 ± 135.73 cm) comparable to the control group, indicating that the combined delivery of curcumin and piperine via the nanoformulation effectively mitigated cuprizone-induced motor impairments. Treatment with the nanoformulation containing curcumin alone (CFZ) did not confer significant protection; these mice exhibited a significantly lower TDM compared to controls (*p* ≤ 0.01). This suggests that curcumin alone, despite its known neuroprotective properties, might be insufficient under the current experimental conditions, possibly due to limited bioavailability or the severity of cuprizone toxicity. The addition of piperine, known to enhance curcumin absorption and efficacy, appears crucial for achieving neuroprotection in this model.

Importantly, after cessation of cuprizone exposure at week five, TDM values in all treatment groups improved and eventually approximated those of the control group, indicating partial or full recovery of motor functions over time. However, the CPZ group, which underwent spontaneous remyelination without any therapeutic intervention, showed a delayed recovery in TDM, highlighting the need for active therapeutic strategies to promote functional recovery post-demyelination.

These observations highlight the potential of combined curcumin–piperine nanoformulations as effective modulators of motor deficits in demyelination models, while also suggesting an unexpected supportive role of the blank formulation, likely linked to the intrinsic properties of the carrier oil (*Z. rhetsa* seed oil).

### 2.3. Effects of Cuprizone and Nanoformulations on Mean Velocity

**Figure 4:**
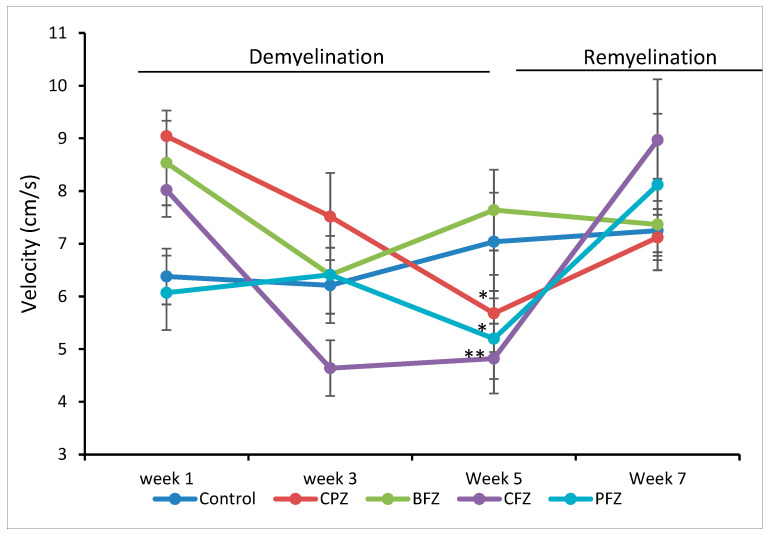
Velocity as determined in Open Field Test. At week 5 significant decrease in velocity in groups CPZ, BFZ and PFZ were seen compared to the velocity in control group. However, the velocity appeared restored at the end of week 7. One-way ANOVA with Tukey’s multiple comparison test was performed to compare the variance between groups. * *p* ≤ 0.05, ** *p* ≤ 0.01.

In line with the findings on total distance moved, cuprizone administration also led to a significant reduction in the mean velocity of mice at the end of the five-week demyelination period. The mean velocity of mice in the CPZ group (5.68 ± 0.73 cm/s; *p* ≤ 0.05), the curcumin-only nanoformulation group (CFZ, 4.82 ± 0.66 cm/s; *p* ≤ 0.01), and the curcumin–piperine nanoformulation group (PFZ, 5.20 ± 0.78 cm/s; *p* ≤ 0.05) was significantly lower than that observed in the control group (7.04 ± 0.93 cm/s) (Figure 4). These reductions reinforce the notion that cuprizone-induced demyelination impairs not only the extent of movement but also the efficiency and speed of locomotion. Interestingly, the blank formulation group (BFZ) maintained a mean velocity comparable to that of the control group, again suggesting a protective or stabilizing influence of the carrier oil. Following the cessation of cuprizone administration after week five, mean velocity values progressively recovered across all groups. Notably, by the end of the recovery period, the mean velocities in the CFZ (8.97 ± 1.15 cm/s) and PFZ (8.12 ± 1.35 cm/s) groups slightly surpassed those of the control (7.25 ± 0.41 cm/s) and CPZ (7.12 ± 0.43 cm/s) groups (Figure 4). This observation suggests that continued treatment with nanoformulations, particularly those containing curcumin alone or in combination with piperine, may not only assist in restoring motor function but potentially enhance locomotor efficiency beyond baseline levels. These improvements could be attributed to enhanced remyelination, reduced neuroinflammation, or upregulated neurotrophic support prompted by antioxidant and anti-inflammatory mechanisms of the therapeutic compounds. Further biochemical and histological analyses are warranted to elucidate the exact mechanisms driving this enhanced functional recovery.

### 2.4. Effects of Cuprizone and Nanoformulations on Grip Strength

**Figure 5:**
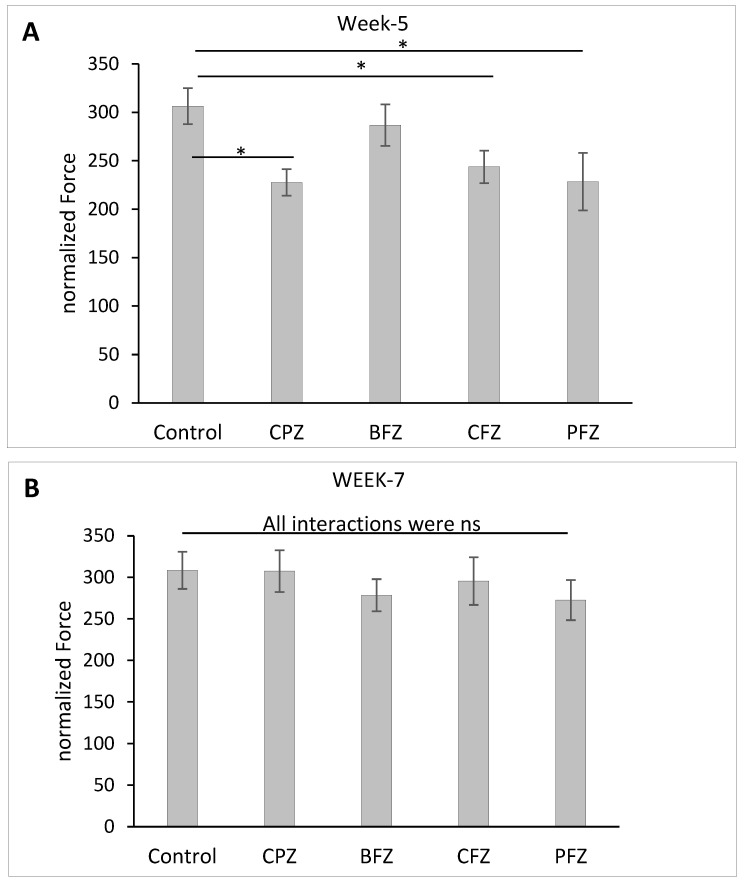
Grip strength expressed as normalized force and determined: (**A**) at week 5 (demyelination); and (**B**) at week 7 (remyelination). One-way ANOVA with Tukey’s multiple comparison test was performed to compare the variance between groups. * *p* ≤ 0.05, ns = nonsignificant.

To further assess motor function, grip strength was evaluated following five weeks of cuprizone feeding. As shown in Figure 5A, the normalized grip strength force was significantly reduced (*p* ≤ 0.05) in the CPZ group as well as in mice treated with the curcumin-only (CFZ) and curcumin–piperine (PFZ) nanoformulations compared to the control group. The grip strength values of the PFZ and CFZ groups were comparable to those of the CPZ group, indicating that, despite their known neuroprotective potential, these formulations were ineffective in preserving neuromuscular strength against cuprizone-induced toxicity during the demyelination phase. The blank formulation group (BFZ) displayed a normalized grip strength (286.73 ± 21.39) similar to that of the control group (306.31 ± 18.59), suggesting that components of the seed oil carrier may confer protective effects, potentially through antioxidant or anti-inflammatory mechanisms that support neuromuscular function. Following the discontinuation of cuprizone administration, grip strength showed significant recovery across all groups. By two weeks post-cuprizone withdrawal, the normalized grip strength force in all treated groups had returned to levels comparable to those of the control group (Figure 5B). This recovery indicates that the neuromuscular impairments induced by cuprizone were largely reversible and that the ongoing nanoformulation treatments may have supported this regenerative process. These findings, together with the data from total distance moved and mean velocity, highlight the complex and variable effects of different nanoformulations on motor function during and after cuprizone-induced demyelination. Curcumin and curcumin–piperine formulations were insufficient in protecting grip strength during active demyelination.

### 2.5. Effects of Cuprizone and Nanoformulations on Motor Coordination and Balance

**Figure 6:**
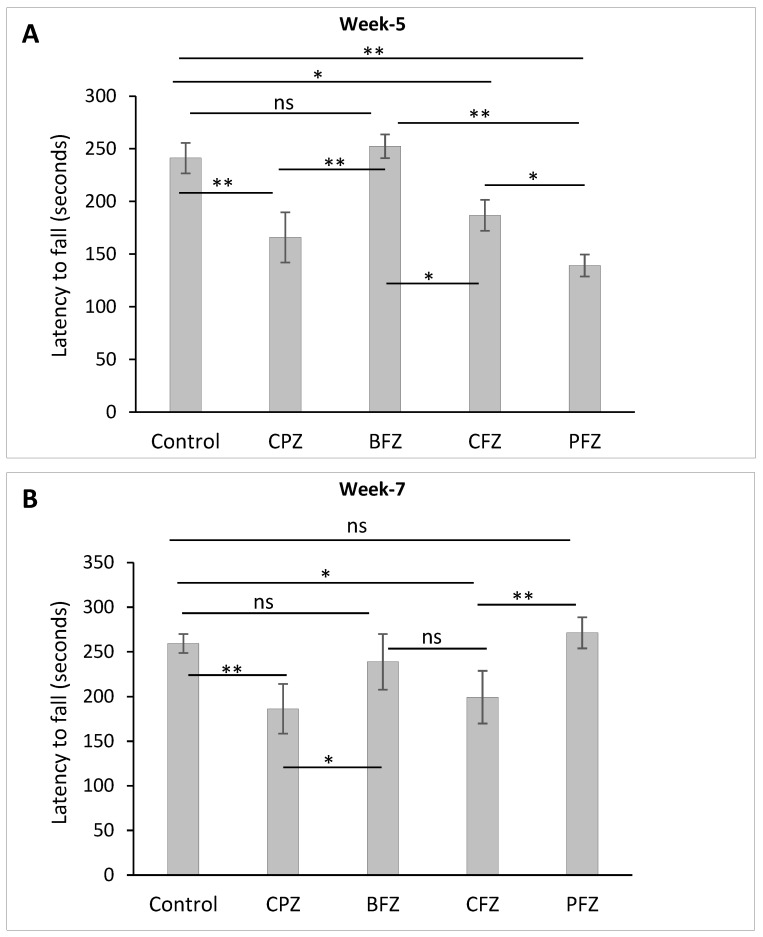
Latency to Fall as determined using rotarod test: (**A**) at the end of week 5 (demyelination) and (**B**) at the end of week 7 (remyelination). One-way ANOVA with Tukey’s multiple comparison test was performed to compare the variance between groups. * *p* ≤ 0.05, ** *p* ≤ 0.01, ns = nonsignificant.

Motor coordination and balance were assessed using the accelerated rotarod test, with latency to fall (measured in seconds) serving as the primary outcome measure. After five weeks of cuprizone feeding, a significant reduction (*p* ≤ 0.01) in latency to fall was observed in the CPZ group compared to the control group, indicating impaired motor coordination and balance resulting from cuprizone-induced demyelination (Figure 6A). Similarly, groups receiving curcumin-only (CFZ) and curcumin–piperine (PFZ) nanoformulations alongside cuprizone also exhibited significantly reduced latencies to fall at week 5. This suggests that these nanoformulations, although potentially neuroprotective in other aspects, were not able to prevent the cuprizone-induced deficits in motor coordination during the active phase of demyelination. In contrast, the blank formulation group (BFZ) demonstrated a latency to fall comparable to that of the control group, reinforcing the idea that the seed oil-based carrier itself may possess beneficial properties capable of preserving neuromotor function under toxic conditions.

Following the cessation of cuprizone exposure after week 5, a gradual recovery in motor performance was noted. By week 7, the PFZ group’s latency to fall had fully recovered to levels similar to those of the control group (Figure 6B), suggesting an effective role of the curcumin–piperine nanoformulation in promoting functional recovery post-demyelination. However, the CPZ group, which underwent spontaneous remyelination without any therapeutic intervention, continued to display significantly shorter latency times compared to controls, indicating persistent deficits in motor coordination and incomplete spontaneous recovery. Collectively, these results highlight the significant impairments in balance and coordination caused by cuprizone, and emphasize the potential of nanoformulations particularly those containing both curcumin and piperine to aid in functional recovery when used in a therapeutic context following demyelination.

### 2.6. Effects of Nanoformulations on Antioxidants Levels

When compared to the control and cuprizone groups, the nanoformulations utilized in this investigation had a significant impact on the levels of CAT, SOD, GSH, and GPX in the frontal brain area at the conclusion of the demyelination stage (Table 2). One well-known copper chelator that causes inflammation and demyelination in the brain is cuprizone. When compared to the CAT level of the control group, significant decreases in CAT levels were seen in groups such as CPZ, BFZ, and CFZ, indicating that neither the blank nor the nanoformulation including curcumin but excluding piperine had any positive impact on CAT levels. The *Z. rhetsa* seed oil nanoformulation including piperine and curcumin (PFZ) showed a notably greater level of catalase when compared to the other groups, suggesting that piperine enhances the bioavailability of curcumin. The SOD level in the CPZ group was substantially lower (*p* ≤ 0.01) than the SOD level in the control group at the conclusion of the demyelination stage (week 5). The SOD levels in BFZ and CFZ were discovered to be considerably lower than the SOD level in the control group, indicating that curcumin was unable to prevent cuprizone poisoning in the frontal cortex in a preventive manner. But when piperine was added to the nanoformulation (PFZ), the level of SOD improved and was comparable to the control group. This showed that the frontal cortex was shielded from the harmful effects of cuprizone by the inclusion of piperine in the nanoformulation. The GSH level in the CPZ group was found to be considerably lower (*p* ≤ 0.0001) than that of the control group (111.65 ± 4.05 ng/mg protein), measuring 57.28 ± 3.13 ng/mg protein. However, at the end of the demyelination stage, it was discovered that the PFZ group had levels of GSH that were similar to those of the control group suggesting thereby the potent effect of curcumin–piperine combination against cuprizone toxicity (Table 2). By the conclusion of the fifth week, it was noticed that the GPX levels in the CPZ, BFZ, and CFZ groups were considerably lower than those in the control group. It is noteworthy that the GPX level (3.46 ± 0.34 ng/mg protein) in the PFZ group was found to be similar to that of the control group (4.34 ± 0.91 ng/mg protein).

Cuprizone was removed from all groups’ feeds at the conclusion of the demyelination stage, which occurred in week five. While BFZ, CFZ, and PFZ continued to receive their respective nanoformulations, the CPZ group was permitted to remyelinate spontaneously. In contrast to the CAT levels in these groups at the conclusion of the demyelination stage, the CPZ, BFZ, and PFZ groups increased in CAT level after cuprizone was terminated in feed (Table 2). The PFZ group had a CAT level that was substantially greater than that of the control group (*p* ≤ 0.001). In comparison to the other groups, the PFZ formulation’s level of SOD was much higher due to the addition of piperine to curcumin. SOD levels in the BFZ, CFZ, and CPZ groups (spontaneous remyelination) did not increase when cuprizone was removed from the rat chow. When comparing the GSH levels of the CPZ, CFZ, and PFZ groups to those of the control group, a substantial rise was seen indicating positive effects of various treatments on GSH levels. When the GPX levels of the demyelinated and remyelinated stages were compared, it was found that the GPX levels in the BFZ and CFZ rose near the conclusion of the former stage. When comparing the GPX level in the CPZ group to its level at week 5, no appreciable rise was seen.

### 2.7. Effects of Nanoformulations on MBP Levels

**Figure 7:**
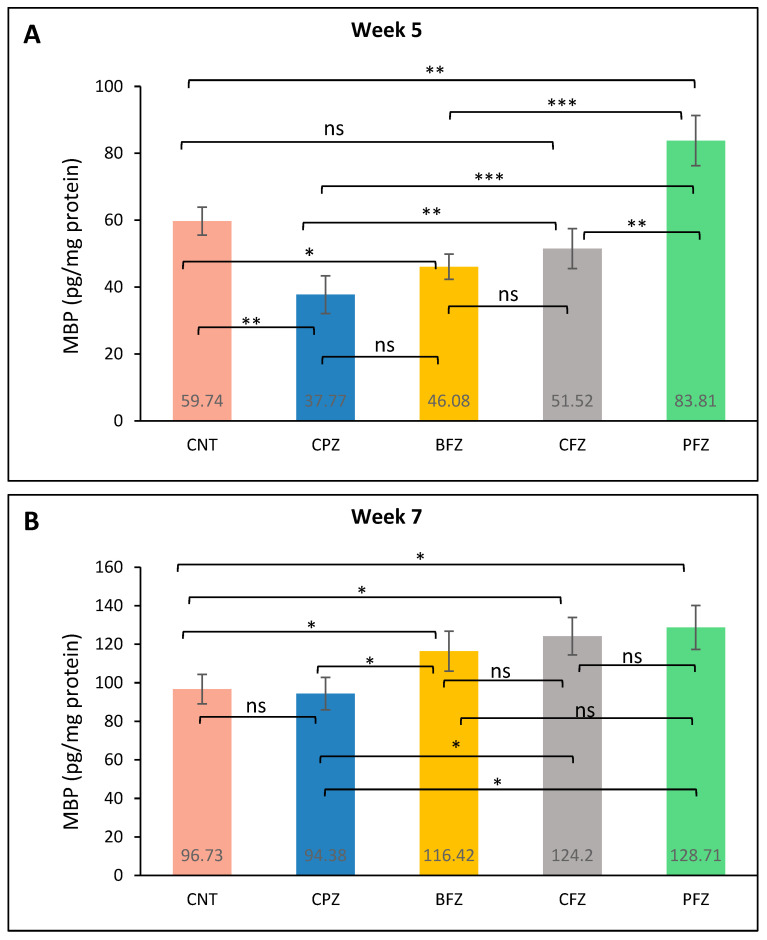
MBP level in different groups: (**A**) at the end of week 5 (demyelination); and (**B**) at the end of week 7 (remyelination). One-way ANOVA with Tukey’s multiple comparison test was performed to compare the variance between groups. * *p* ≤ 0.05, ** *p* ≤ 0.01, *** *p* ≤ 0.001, ns = nonsignificant.

At the conclusion of week 5, MBP levels in the frontal cortex were significantly reduced in the cuprizone-treated group (37.77 ± 5.64 pg/mg protein) compared to the control group (59.74 ± 4.21 pg/mg), confirming demyelination (Figure 7A). Among the treatment groups, the PFZ nanoformulation showed the highest restorative effect, elevating MBP levels to 83.81 ± 7.48 pg/mg, surpassing even the control. The CFZ group demonstrated moderate protection with MBP levels at 51.52 ± 5.94 pg/mg, followed by the BFZ group at 46.08 ± 3.78 pg/mg. These findings suggest that PFZ most effectively counteracted CPZ-induced myelin loss in the frontal cortex. By the end of week 7, following cessation of CPZ treatment at week 5, MBP levels in the frontal cortex showed partial recovery in the CPZ-only group (Figure 7B), now going on spontaneous recovery, (94.38 ± 8.42 pg/mg protein), approaching the CNT group value (96.73 ± 7.56 pg/mg). Notably, continued administration of curcumin nanoformulations led to further increases in MBP levels, suggesting enhanced remyelination. The PFZ group demonstrated the most pronounced effect, reaching 128.71 ± 11.46 pg/mg, followed by CFZ (124.20 ± 9.72 pg/mg) and BFZ (116.42 ± 10.38 pg/mg). These findings indicate that post-demyelination treatment with nanoformulations, particularly PFZ, significantly promotes remyelination beyond spontaneous recovery. These results strongly suggest that curcumin nanoformulations, particularly PFZ and CFZ, substantially promote remyelination following CPZ-induced demyelination, outperforming the natural recovery observed in the CPZ-only group.

### 2.8. Effects of Nanoformulations on AChE Levels

**Figure 8:**
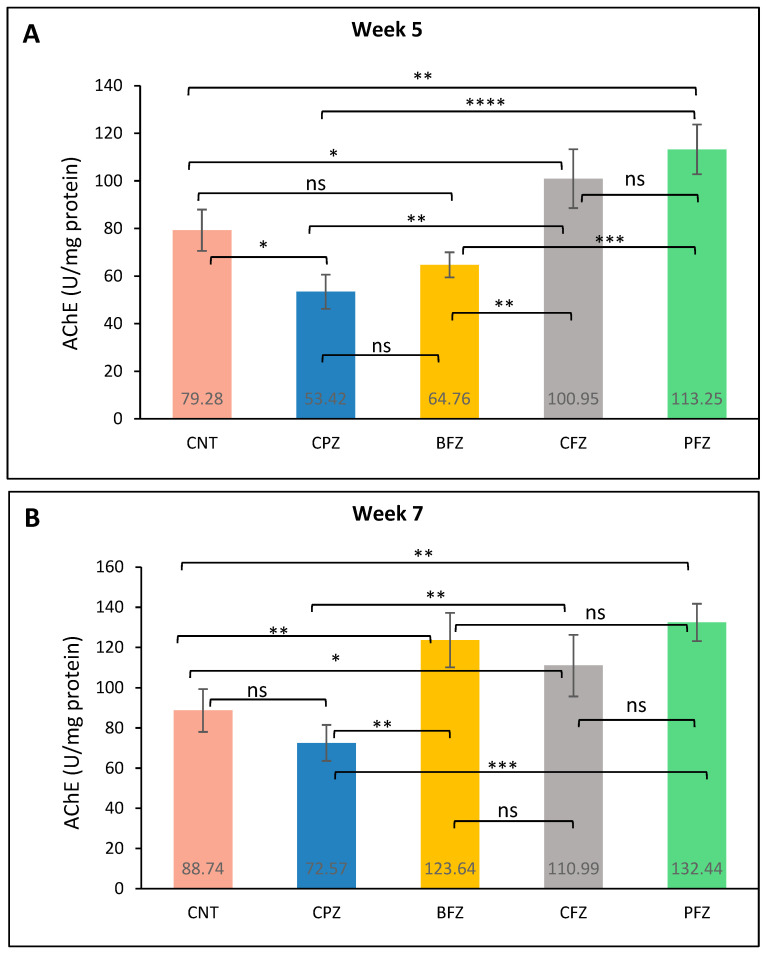
AChE level in different groups: (**A**) at the end of week 5 (demyelination), and (**B**) at the end of week 7 (remyelination). One-way ANOVA with Tukey’s multiple comparison test was performed to compare the variance between groups. * *p* ≤ 0.05, ** *p* ≤ 0.01, *** *p* ≤ 0.001, **** *p* ≤ 0.0001; ns = nonsignificant.

At week 5, acetylcholinesterase (AChE) levels in the frontal cortex were significantly reduced in the CPZ group (53.42 ± 7.16 U/mg protein) compared to the control group (79.28 ± 8.68 U/mg protein), indicating impaired cholinergic function due to cuprizone-induced neurotoxicity (Figure 8A). Treatment with curcumin nanoformulations resulted in varying degrees of restoration. The PFZ group exhibited the most substantial increase in AChE levels (113.25 ± 10.44 U/mg protein), exceedingly even the control value. CFZ also showed a strong effect (100.95 ± 12.38 U/mg protein), followed by BFZ with a moderate improvement (64.76 ± 5.24 U/mg protein). These results suggest that the PFZ and CFZ formulations effectively counteracted CPZ-induced cholinergic disruption and enhanced AChE activity. At the conclusion of week 7, AChE levels in the CPZ-only group (72.57 ± 8.94 U/mg protein) showed partial recovery following the cessation of cuprizone treatment (Figure 8B), though levels remained below those of the control group (88.74 ± 10.66 U/mg protein). In contrast, continued administration of curcumin nanoformulations led to a pronounced increase in AChE activity. The PFZ-treated group exhibited the highest level (132.44 ± 9.28 U/mg protein), followed by BFZ (123.64 ± 13.56 U/mg protein) and CFZ (110.99 ± 15.24 U/mg protein), all of which exceeded control values. These findings indicate that the nanoformulations, particularly PFZ and BFZ, not only reversed CPZ-induced cholinergic suppression but also significantly enhanced AChE activity beyond baseline levels, potentially reflecting improved cognitive function and synaptic integrity.

### 2.9. Effects of Nanoformulations on CD4 and CD8 Levels

At week 5, the CPZ group (18,466.57 ± 162.34 pg/mg protein) exhibited a dramatic and statistically significant (*p* ≤ 0.0001) increase in CD4 expression compared to the CNT group (2725.25 ± 213.66 pg/mg protein), indicating pronounced cuprizone-induced neuroinflammation (Table 3). Treatment with nanoformulations significantly attenuated CD4 levels in BFZ, CFZ and PFZ groups. When compared to the control, the BFZ group showed a mild but significant elevation (*p* ≤ 0.01), whereas CFZ and PFZ were also slightly elevated compared to CNT (*p* ≤ 0.05), though much lower than the CPZ group. No significant difference was observed between CFZ and PFZ. These findings suggest that all nanoformulations mitigated CPZ-induced CD4 elevation, with PFZ showing the most pronounced reduction, bringing levels closest to the control. At conclusion of Week 7, CD4 levels remained markedly elevated in the CPZ group (17,071.16 ± 138.48 pg/mg protein) compared to the CNT (2585.69 ± 231.56 pg/mg protein). Although cuprizone was stopped after Week 5 and this group undergone spontaneous recovery but still showing a sustained neuroinflammatory effect from cuprizone administration until Week 5 (*p* ≤ 0.0001). Nanoformulation treatments resulted in significant reductions in CD4 expression compared to the CPZ group. Compared to CNT, BFZ-treated animals still showed slightly elevated CD4 levels (*p* ≤ 0.01), while CFZ and PFZ showed significant reductions below control levels. These results suggest that two weeks into the stopping of cuprizone resulted in the reversal of cuprizone-induced elevation in CD4 levels in PFZ and CFZ even lower than baseline, indicating a potentially strong immunosuppressive or regulatory effect. BFZ continued to reduce CD4 expression compared to CPZ, though to a lesser extent than CFZ and PFZ.

Following five weeks of cuprizone administration, CD8 expression was significantly elevated in the CPZ group compared to the CNT group (*p* ≤ 0.0001), indicating enhanced cytotoxic T-cell activity and a strong neuroinflammatory response. Treatment with nanoformulations effectively attenuated CD8 levels (Table 3). Compared to CNT, CFZ and PFZ had CD8 levels slightly lower than the control (*p* ≤ 0.05), suggesting a potential immunoregulatory effect. At week 7, CD8 expression in the CPZ group (5.86 ± 0.74 pg/mg protein) remained slightly elevated compared to the control group (5.18 ± 0.42 pg/mg protein); however, the difference was minimal, indicating a partial spontaneous recovery following the cessation of cuprizone exposure. Treatment with nanoformulations further reduced CD8 levels. CFZ and PFZ reduced CD8 expression below baseline, suggesting an enhanced suppressive effect. These findings indicate that while spontaneous recovery following CPZ withdrawal led to partial normalization of CD8 expression, nanoformulations particularly PFZ and CFZ induced further reductions, potentially reflecting a continued immunomodulatory effect that may limit residual cytotoxic T-cell activity and support resolution of inflammation.

### 2.10. Effects of Nanoformulations on the mRNA Levels of BDNF, CREB, TNFα, and IL-1β

**Figure 9:**
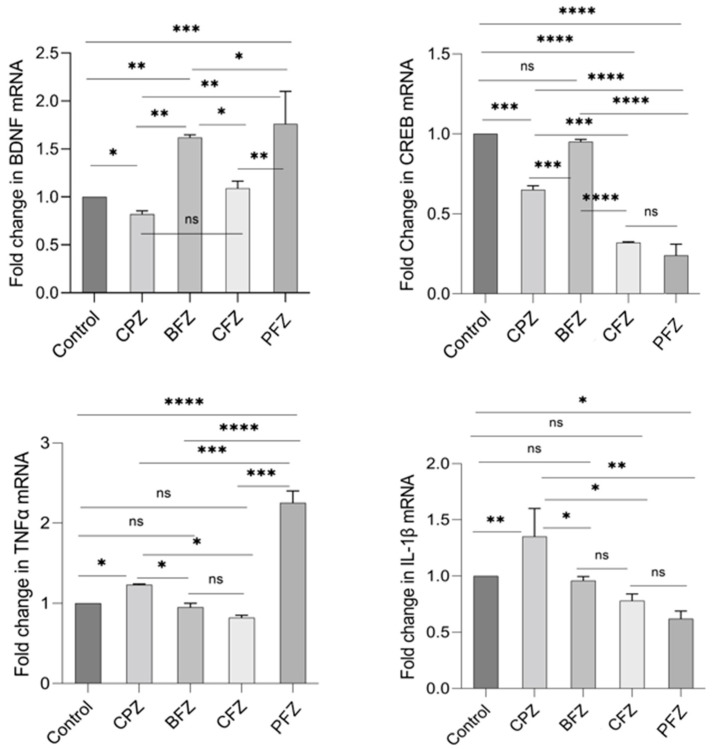
Fold change in mRNA expression levels of BDNF, CREB, TNFα, and IL-1β in frontal cortex at the end of demyelination stage. One-way ANOVA with Tukey’s multiple comparison test was performed to compare the variance between groups. * *p* ≤ 0.05, ** *p* ≤ 0.01, *** *p* ≤ 0.001, **** *p* ≤ 0.0001; ns = nonsignificant.

Quantitative real-time PCR (qRT-PCR) was employed to assess the mRNA expression levels of BDNF, CREB, TNFα, and IL-1β. By the end of week 5, BDNF mRNA expression in the CPZ group was significantly reduced compared to the control group (*p* ≤ 0.05; Figure 9). In contrast, BDNF mRNA expression was significantly elevated in the BFZ, CFZ, and PFZ groups relative to both the control and CPZ groups (Figure 9). CREB mRNA expression was also markedly reduced in the CPZ group at the conclusion of the demyelination phase (*p* ≤ 0.001) compared to the control. Moreover, the CFZ and PFZ groups exhibited significantly lower CREB mRNA levels compared to both the CPZ and control groups. Notably, the BFZ group maintained CREB mRNA expression levels similar to the control, demonstrating resistance to the CPZ-induced decline. Regarding TNFα, mRNA expression in the BFZ and CFZ groups did not significantly differ from the control. However, TNFα mRNA levels were significantly elevated in the PFZ group (*p* ≤ 0.0001) and the CPZ group (*p* ≤ 0.05) compared to the control. By the end of week 5, IL-1β mRNA expression was also significantly increased relative to the control group (*p* ≤ 0.01), although reductions were observed in some treatment groups (Figure 9).

**Figure 10:**
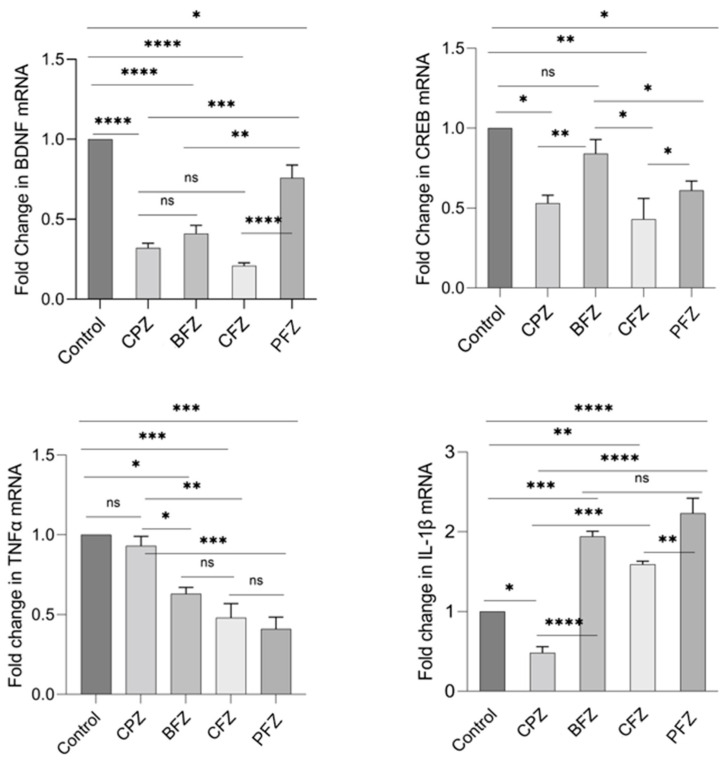
Fold change in mRNA expression levels of BDNF, CREB, TNFα, and IL-1β in frontal cortex at the end of remyelination stage. One-way ANOVA with Tukey’s multiple comparison test was performed to compare the variance between groups. * *p* ≤ 0.05, ** *p* ≤ 0.01, *** *p* ≤ 0.001, **** *p* ≤ 0.0001; ns = nonsignificant.

At the conclusion of the remyelination phase (week 7), following the withdrawal of cuprizone, BDNF mRNA levels in the CPZ group continued to decline, with levels even lower than those observed at the demyelination stage. BDNF mRNA expression in the BFZ, CFZ, and PFZ groups remained significantly lower than that of the control group (Figure 10). The mRNA expression pattern of CREB mirrored that of BDNF, with continued suppression in the CPZ, CFZ, and PFZ groups by week 7. In contrast, TNFα mRNA expression was significantly reduced in the BFZ, CFZ, and PFZ groups relative to their levels at the end of the demyelination phase. In the CPZ group, TNFα mRNA levels returned to values comparable to the control. Similar trends were observed for IL-1β mRNA expression, which paralleled changes seen in TNFα expression.

### 2.11. Effects of Nanoformulations on the Protein Levels of BDNF, CREB, TNFα, and IL-1β

At the conclusion of the demyelination phase, BDNF levels in the CPZ group were significantly reduced compared to the control group, showing a 26% decrease. As presented in Table 4, BDNF levels in the BFZ and PFZ groups were significantly elevated (*p* ≤ 0.01), with increases of 47.79% and 57.49%, respectively, relative to the control. In contrast, BDNF levels in the CFZ group (curcumin without piperine) remained comparable to those of the control. Similarly, CREB levels in the CPZ group were markedly decreased by 30.87% (*p* ≤ 0.01) relative to the control. The BFZ group preserved CREB levels comparable to those of the control, whereas the CFZ and PFZ groups exhibited substantial reductions of 74.52% and 78.37%, respectively. TNFα levels were significantly elevated in the CPZ group compared to the control (*p* ≤ 0.01). However, both CFZ and BFZ groups exhibited modestly lower TNFα levels (312.41 ± 23.25 and 347.46 ± 16.52 pg/mg protein, respectively) relative to the control (373.03 ± 13.67 pg/mg protein). In contrast, the PFZ group demonstrated significantly higher TNFα levels compared to the CPZ group (*p* ≤ 0.0001). By the end of week 5, IL-1β levels in the CPZ group increased by 31.59% relative to the control (*p* ≤ 0.0001). Notably, the BFZ, CFZ, and PFZ groups effectively maintained IL-1β levels significantly lower than those observed in both the control and CPZ groups (Table 4).

Following the remyelination phase at week 7, BDNF levels in the CPZ group—despite the cessation of cuprizone after week 5 remained 53% lower than those in the control group (*p* ≤ 0.0001). Although BDNF levels in the BFZ, CFZ, and PFZ groups remained below control values, they showed notable improvements relative to the CPZ group (Table 5). In terms of CREB, no significant recovery was observed in the CPZ, CFZ, or PFZ groups following cuprizone withdrawal. However, CREB levels in the BFZ group (839.46 ± 44.39 pg/mg protein) were comparable to those of the control group (917.69 ± 38.53 pg/mg protein). Upon cuprizone removal, TNFα levels in the CPZ group returned to levels comparable to the control. Conversely, TNFα levels were significantly lower in the nanoformulation-treated groups (BFZ, CFZ, and PFZ) compared to both the control and CPZ groups, with reductions of approximately 50% observed in the CFZ and PFZ groups (Table 5). Finally, at the end of the remyelination phase, IL-1β levels, which had been markedly elevated during demyelination, significantly decreased (*p* ≤ 0.001) relative to the control. Interestingly, IL-1β levels were significantly higher in the nanoformulation-treated groups (BFZ, CFZ, and PFZ) compared to both the control and CPZ groups (Table 5).

### 2.12. Histological Examinations

**Figure 11:**
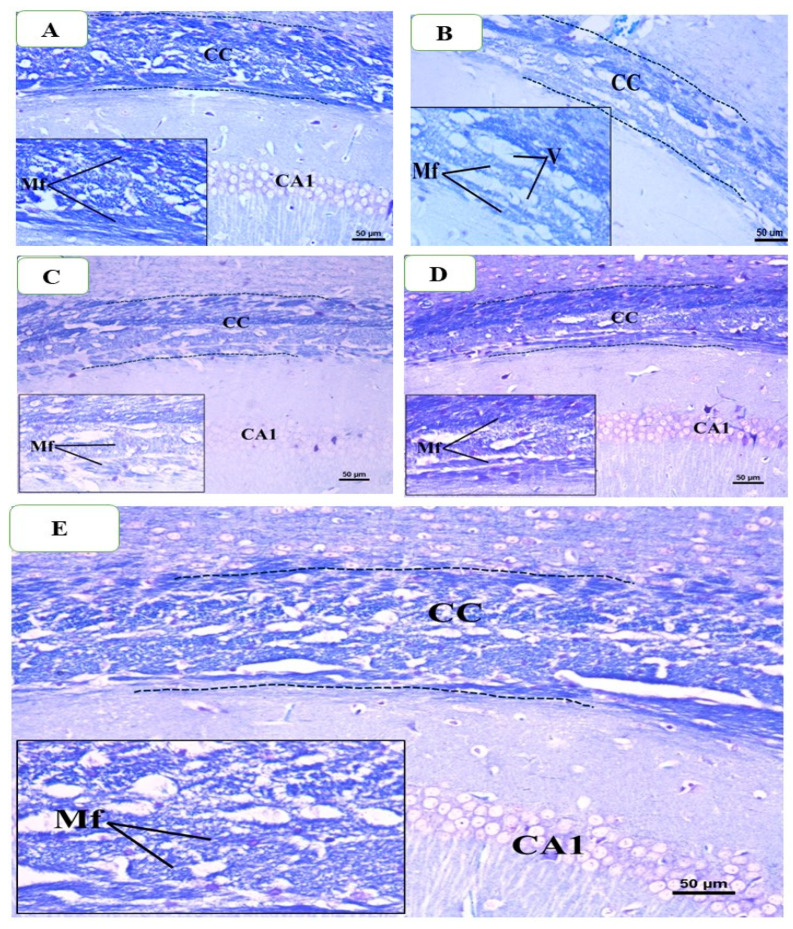
Representative photomicrographs of the corpus callosum (CC) from different groups at the end of week 5: (**A**) Control group showing normal dense blue staining intensity and normal myelinated fibers (Mf) in the CC. (**B**) Cuprizone treated group showing significant reduction in staining intensity in the above areas as compared to control and decreased myelinated nerve fibers and fewer oligodendrocytes of small size. Moreover, many vacuoles are also visible (**C**) The BFZ group (treated with CPZ + ZRO) showed mild increase in staining intensity as compared to the CPZ group with the presence of some demyelinated nerve fibers. (**D**) The CFZ group (CPZ + curcumin + ZRO) showed moderate increase in staining intensity as compared to the CPZ and BFZ groups with the presence of some demyelinated nerve fibers. (**E**) The PFZ group (CPZ + curcumin + piperine + ZRO) showed less better staining as well as myelinated fibers compared to BFZ, and CFZ groups. Also, more numerous vacuoles were seen than BFZ and CFZ group. Magnification Original image: ×200; Insets: ×400.

Under a microscope, brain samples from the control group showed typical corpus callosum (CC) histology, which is characterized by parallel, tightly packed, myelinated fibers that stain dark blue when using Luxor fast blue (LFB). As seen in Figure 11A, the oligodendrocyte nuclei were rounded, well-defined, darkly pigmented, and positioned in between the myelinated fibers. However, a significant decrease in the intensity of CC’s LFB staining was seen in brain slices from the CPZ-treated group after five weeks of treatment as compared to the control group. The CC nerve fibers in the CPZ group had several regions of demyelination, fragmentation, and disarray. Also, the nuclei of numerous oligodendrocytes showed signs of dispersion, dark staining, and condensation, whereas the nuclei of other cells showed very weak staining (Figure 11B). Examining the BFZ group (given CPZ + ZRO), it was found that numerous demyelinated and unpacked nerve fibers were present, along with many darkly stained, condensed oligodendrocyte nuclei (Figure 11C) and a decrease in the intensity of LFB staining of CC. Many of the CC nerve fibers in the CFZ group, which received CPZ and curcumin ZRO, had decent myelination and were densely packed with moderately intense LFB staining. Additionally, a large number of oligodendrocyte nuclei seemed to be intact and positioned in between the nerve fibers (Figure 11D). Examining the PFZ group (CPZ + curcumin + piperine + ZRO) showed that the nerve fibers were mostly myelinated and had a normal color intensity, much like the control group. Additionally, the oligodendrocytes seemed to be intact and positioned between the nerve fibers (Figure 11E).

**Figure 12:**
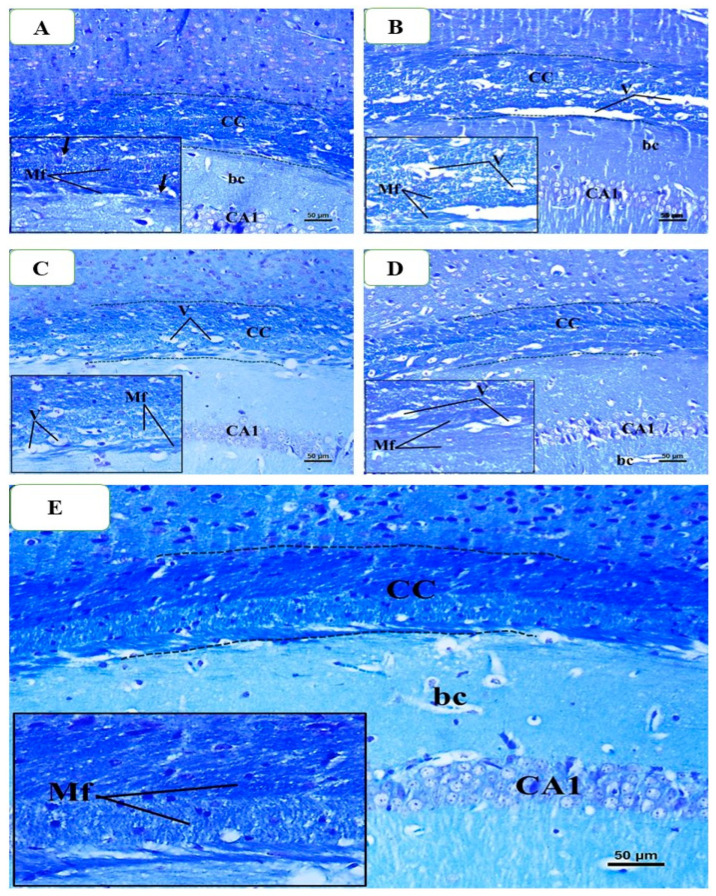
Representative photomicrographs of the corpus callosum (CC) from different groups at the end of week 7: (**A**) Control group showed normal dense blue staining intensity and densely packed, myelinated fibers in the CC. (**B**) The CPZ group (no treatment after week 5) exhibited a similar staining pattern as of control; however, there were still many areas of demyelination, fragmentation, and disorganization of CC nerve fibers. (**C**) The BFZ group (treated with ZRO after week 5) showed nearly similar staining intensity compared to control group. (**D**) The CFZ group (curcumin + ZRO) showed improved staining intensity as compared to the CPZ group. (**E**) The PFZ group (curcumin + piperine + ZRO) appears to restore normal dense blue staining intensity and densely packed, myelinated fibers in the CC. Magnification Original image: ×200; Insets: ×400.

The histological analysis of LFB-stained slides was once more performed on brain samples taken from mice that were killed at the conclusion of the seventh week. When employing LFB, the control group’s histology revealed typical CC, which is defined by parallel, densely packed, myelinated fibers that stain dark blue. The oligodendrocyte nuclei were positioned in the space between the myelinated fibers (Figure 12A). In contrast, there were significant reductions in the color intensity of LFB staining in the CPZ-treated group compared to the control group after week 5, and there were still numerous areas of demyelination, fragmentation, and disarray of CC nerve fibers with the appearance of large vacuoles and spaces between them (Figure 12B). A little improvement was seen when comparing the BFZ group to the CPZ group, as evidenced by the existence of a few vacuoles, many demyelinated and unloaded nerve fibers, and a decrease in the intensity of LFB staining of CC (Figure 12C). In contrast, a large number of the CC nerves in the CFZ group exhibited satisfactory myelination and were packed tightly with fairly high LFB staining. But there were also some demyelinated and disorganized nerve fibers, as well as a small number of vacuoles (Figure 12D). Most of the nerve fibers in the PFZ group were myelinated, and they looked and had the same intensity of color as the control group (Figure 12E).

## 3. Discussion

The present study demonstrates that cuprizone-induced demyelination leads to profound motor impairments, as evidenced by reductions in total distance moved, mean velocity, grip strength, and motor coordination in the rotarod test. These findings align with previous studies reporting cuprizone as a robust model for mimicking the motor and cognitive deficits associated with multiple sclerosis and other demyelinating conditions [5,6]. The observed deficits reflect both the loss of myelin integrity and the consequent disruption of neuronal signaling pathways critical for motor control. Importantly, the use of curcumin and curcumin–piperine nanoformulations revealed differential protective and restorative effects. While neither formulation fully prevented motor impairments during active cuprizone exposure, notable improvements in motor outcomes were observed following cuprizone withdrawal, particularly in the curcumin–piperine group. Piperine is known to enhance the bioavailability of curcumin by inhibiting its metabolic breakdown [30], which likely accounts for the superior recovery of motor function observed in the PFZ group compared to curcumin alone.

The blank formulation (BFZ) also demonstrated remarkable protective effects across all motor tests, maintaining performance at levels comparable to controls even during active cuprizone exposure. This suggests that the carrier oil used in the nanoformulation, derived from *Zanthoxylum rhetsa* seeds, possesses intrinsic antioxidant and anti-inflammatory properties, which may counteract cuprizone toxicity. Previous studies have indicated that plant-derived oils rich in bioactive lipids can modulate oxidative stress and inflammation [19], which are central mechanisms in demyelination pathology.

The predominance of oleic acid and palmitic acid as determined through GC-MS highlights the *Z. rhetsa* seed oil’s similarity to other nutritionally valuable oils, such as olive and peanut oil [31]. Oleic acid is a monounsaturated fatty acid associated with cardioprotective, antioxidant, and anti-inflammatory effects, while palmitic acid contributes to metabolic and structural roles in cell membranes. The combined abundance of these fatty acids suggests that *Z. rhetsa* seed oil may hold potential as a functional dietary oil. The presence of hexanal, nonanal, 2,4-decadienal, and trans-2-undecenal indicates contributions to the organoleptic properties of the oil. These aldehydes are known for green, fatty, and citrus-like odor notes, making them important aroma-active compounds [32]. Their detection highlights the potential role of *Z. rhetsa* seed oil in culinary applications and flavoring industries. Although present in small quantities, 4-methylphenol, oxacyclotetradecane-2,11-dione, and dihexylsulfide may enhance the oil’s bioactive profile. Phenolic compounds such as 4-methylphenol possess antimicrobial activity [33], while sulfur compounds and lactones are often associated with antimicrobial, preservative, and fragrance properties. Even at low concentrations, these compounds can significantly influence therapeutic and industrial applications.

The chemical profile of *Z. rhetsa* seed oil differs significantly from the oils extracted from pericarp and leaves or fruit of related species (*Z. mantaro*, *Z. armatum*), which are dominated by monoterpenes and sesquiterpenes [34,35]. This contrast highlights a chemotypic distinction between plant parts: pericarp oils serve primarily as aromatic/essential oils, while seed oils are fatty acid-rich and may serve nutritional, nutraceutical and medicinal use as an antimicrobial or antioxidant formulation.

The partial reversibility of motor deficits after cuprizone cessation further emphasizes the dynamic nature of demyelination and remyelination processes in the CNS. Nevertheless, the delayed and incomplete recovery observed in the untreated CPZ group highlights the necessity of therapeutic intervention to enhance functional restoration. Overall, these results suggest that nanoformulations combining curcumin and piperine offer promising therapeutic potential for mitigating motor dysfunction in demyelinating diseases, potentially through antioxidant, anti-inflammatory, and neurotrophic mechanisms.

Oxidative stress is widely recognized as a key contributor to cuprizone-induced demyelination and neuronal dysfunction. Cuprizone administration leads to excessive production of reactive oxygen species (ROS), overwhelming the endogenous antioxidant defense systems and resulting in lipid peroxidation, mitochondrial dysfunction, and axonal injury [6,36]. In this context, antioxidant enzymes such as catalase (CAT), superoxide dismutase (SOD), glutathione peroxidase (GPX), and glutathione (GSH) play crucial roles in maintaining redox homeostasis and protecting neural tissue integrity. GSH, GPX, CAT and SOD levels were significantly reduced in CPZ and partially restored in PFZ and CFZ, suggesting potent antioxidative recovery with curcumin/piperine combination. The observed motor impairments in the CPZ group likely reflect a failure of these antioxidant defenses under conditions of sustained oxidative burden. Notably, the improvement in motor function following treatment with curcumin–piperine nanoformulations may be attributed, at least in part, to the known antioxidant properties of curcumin and piperine, which have been shown to upregulate endogenous antioxidant enzymes and scavenge free radicals [37]. Furthermore, the protective effects observed in the BFZ group suggest that components of the *Zanthoxylum rhetsa* seed oil may contribute additional antioxidant capacity, thereby mitigate oxidative stress and preserving motor function during cuprizone-induced demyelination.

MBP is essential for maintaining myelin sheath integrity and is a reliable marker of myelination in the CNS [17]. At week 5, significantly reduced MBP levels in the CPZ group confirmed effective demyelination, consistent with prior reports using cuprizone to model multiple sclerosis-like pathology [38]. Among treatments, the PFZ nanoformulation showed the strongest protective effect, elevating MBP above control levels, suggesting enhanced myelin preservation or regeneration. By week 7, MBP levels in the CPZ-only group partially recovered, reflecting spontaneous remyelination after cuprizone withdrawal [39]. However, nanoformulation treatments especially PFZ and CFZ further elevated MBP, surpassing both control and recovery levels, indicating active promotion of remyelination. This effect likely results from curcumin’s known anti-inflammatory, antioxidant, and pro-oligodendrocyte effects, which may be amplified by nanoformulation and piperine co-administration [40].

Acetylcholinesterase (AChE) is a key enzyme responsible for hydrolysing acetylcholine, a neurotransmitter that plays a pivotal role in learning, memory, and overall cognitive processing [41]. Dysregulation of AChE activity is often linked to cognitive impairments and neurodegenerative disorders, including MS, where demyelination and inflammation disrupt cholinergic signaling [42]. In this study, significantly reduced AChE activity observed in the cuprizone (CPZ)-treated group at week 5 is indicative of impaired cholinergic neurotransmission, likely contributing to cognitive deficits commonly associated with CPZ-induced demyelination [43]. By week 7, a partial restoration of AChE activity in the CPZ group suggests some degree of spontaneous recovery of the cholinergic system following cessation of cuprizone. However, animals treated with nanoformulations especially CFZ and PFZ demonstrated significantly higher AChE activity compared to both CPZ and control groups, implying a more robust restoration or enhancement of cholinergic function. These findings align with previous reports that curcumin and its analogs can improve cholinergic activity through antioxidant and anti-inflammatory pathways, thus protecting neurons and supporting synaptic function [44]. The particularly marked upregulation of AChE in the PFZ group which includes piperine, a known bioavailability enhancer—suggests a synergistic neuroprotective effect. This may involve attenuation of oxidative stress and neuroinflammation, both of which are known contributors to cholinergic dysfunction in MS models [45,46]. Enhanced AChE activity in PFZ-treated animals could thus be reflective of improved synaptic integrity and neurotransmitter regulation, which may translate into better cognitive outcomes.

Inflammation plays a critical role in the pathophysiology of cuprizone-induced demyelination, with pro-inflammatory cytokines such as tumor necrosis factor-alpha (TNF-α) and interleukin-1 beta (IL-1β) being central mediators of neuroinflammation and oligodendrocyte injury [47]. In this study, the CPZ group exhibited elevated levels of TNF-α and IL-1β, consistent with previous reports demonstrating the activation of glial cells and subsequent inflammatory cascades in response to cuprizone exposure. Notably, both curcumin and curcumin–piperine nanoformulations were able to reduce these inflammatory markers, suggesting that these formulations exert their neuroprotective effects at least in part through modulation of neuroinflammation. Curcumin’s anti-inflammatory properties are well documented, and its ability to suppress NF-κB signaling, a key pathway in the activation of pro-inflammatory cytokines, may explain its beneficial effects on motor function recovery [48].

In addition to inflammatory cytokines, neurotrophic factors such as brain-derived neurotrophic factor (BDNF) and cAMP response element-binding protein (CREB) play essential roles in neuronal survival, synaptic plasticity, and remyelination. In our study, increased levels of BDNF and CREB signaling were observed in groups treated with nanoformulations, particularly those containing both curcumin and piperine, suggesting enhanced neurotrophic support and improved remyelination. These findings are consistent with the notion that curcumin and its derivatives can upregulate neurotrophic factors and promote neuroprotection and regeneration [14]. The ability of the curcumin–piperine nanoformulation to modulate both inflammatory and neurotrophic pathways likely contributes to the observed restoration of motor function following cuprizone withdrawal.

The current study investigated the effects of cuprizone-induced demyelination and subsequent nanoformulation-based treatments on the expression of T-cell markers CD4 and CD8, which are key indicators of helper and cytotoxic T-cell activity, respectively. The temporal dynamics observed across week 5 and week 7 provide important insights into the neuroimmune response to demyelination and its modulation by therapeutic interventions. At week 5, cuprizone administration resulted in a dramatic upregulation of both CD4 and CD8 expression, consistent with acute neuroinflammatory activation. CD4 levels were nearly seven-fold higher than control values, while CD8 expression also tripled, highlighting the mobilization of both helper and cytotoxic T-cell responses. These findings are in line with previous studies showing that cuprizone-induced demyelination triggers peripheral immune cell infiltration, particularly involving T-helper cells (Th1/Th17) and cytotoxic CD8+ T cells, contributing to CNS damage [49,50]. Nanoformulation treatments (BFZ, CFZ, PFZ) led to significant reductions in both markers, with PFZ consistently demonstrating the most pronounced effect. Notably, PFZ and CFZ brought CD4 and CD8 levels close to or below baseline, suggesting a potent immunoregulatory influence. By week 7, despite cessation of cuprizone exposure, the CPZ group continued to show elevated CD4 levels, indicating persistent neuroinflammation and inadequate spontaneous resolution. CD8 levels, however, had partially normalized, suggesting differential kinetics of T-cell subpopulation recovery or clearance. This residual CD4 elevation supports the idea that helper T-cell–driven inflammation may persist longer, potentially contributing to chronic neuroinflammatory states observed in demyelinating diseases like multiple sclerosis [51]. Remarkably, PFZ and CFZ not only sustained the suppression of both CD4 and CD8, but reduced them below control levels, implying that these nanoformulations exert an ongoing suppressive effect on immune activity even after the inflammatory stimulus has ceased. This could involve inhibition of T-cell activation pathways (e.g., NF-κB, MAPKs), promotion of regulatory T-cell responses, or enhanced clearance of pro-inflammatory cells from the CNS [52]. PFZ, in particular, may have improved targeting efficiency or sustained-release kinetics that enable deeper immune modulation. These results underscore the therapeutic potential of nanoformulations in regulating both arms of the adaptive immune response in demyelinating conditions. While CD4 suppression is critical for dampening pro-inflammatory cytokine release and microglial activation, CD8 modulation may help reduce direct cytotoxic damage to oligodendrocytes and neurons.

Histological analysis, particularly using Luxol Fast Blue (LFB) staining, provides valuable insight into the extent of remyelination and the restoration of myelin integrity following cuprizone-induced demyelination. In this study, LFB staining revealed a clear reduction in myelin staining in the CPZ group, consistent with the observed motor impairments and functional deficits. This reduction in myelination corresponds to the significant decline in total distance moved, velocity, grip strength, and rotarod performance. In contrast, treatment with nanoformulations, especially those containing curcumin and piperine, was associated with more prominent remyelination in regions previously affected by cuprizone toxicity. These histological improvements correlate with the restoration of motor function observed in behavioral tests, supporting the hypothesis that the nanoformulations facilitated remyelination through antioxidant, anti-inflammatory, and neurotrophic mechanisms.

Curcumin exerts its neuroprotective effects through modulation of multiple molecular pathways relevant to neuroinflammation and myelination. It suppresses the activation of NF-κB, a key transcription factor involved in the expression of pro-inflammatory cytokines, thereby attenuating neuroinflammatory responses [53]. Additionally, curcumin activates the Nrf2/ARE signaling pathway, enhancing the expression of endogenous antioxidant enzymes such as HO-1 and SOD, which protect neural tissue from oxidative stress-induced damage [54]. Curcumin also promotes remyelination and neuronal survival by upregulating BDNF and enhancing CREB phosphorylation, both of which are critical for oligodendrocyte function and myelin repair [55]. These pleiotropic actions make curcumin a promising agent for modulating key pathological features of demyelinating diseases like multiple sclerosis.

In the present study, the curcumin–piperine nanoformulation (PFZ) demonstrated the most extensive remyelination compared to other treatment groups, including the curcumin-only (CFZ) formulation and the blank formulation (BFZ). This enhanced remyelination in the PFZ group is likely a result of the synergistic effects of curcumin and piperine in promoting both anti-inflammatory responses and the upregulation of neurotrophic factors like BDNF and CREB, which are crucial for oligodendrocyte survival and myelination [56]. The significant recovery of motor function in this group further underscores the importance of these compounds in supporting myelin repair and functional recovery following neuroinflammation and demyelination.

Several FDA-approved therapies for MS, including fingolimod, glatiramer acetate, dimethyl fumarate, and interferon-β, primarily act through immunomodulatory or immunosuppressive mechanisms to reduce relapse frequency and slow disease progression. For example, fingolimod modulates sphingosine-1-phosphate receptors to sequester lymphocytes in lymph nodes, while glatiramer acetate induces regulatory T-cell responses and shifts cytokine profiles. However, these agents often do not directly promote remyelination or exert neurotrophic effects, and some are associated with significant systemic side effects. In contrast, the PFZ nanoformulation developed in this study not only demonstrated immunomodulatory actions (e.g., reduction in CD4/CD8 levels and inflammatory cytokines) but also significantly enhanced remyelination, antioxidant defenses, and neu.rotrophic factor expression (e.g., BDNF, CREB). Thus, PFZ offers a multimodal therapeutic approach that may complement or surpass current MS treatments by addressing both inflammatory and neurodegenerative aspects of the disease.

### Strengths and Limitations

The study is limited by the absence of mechanistic experiments delineating the pathways modulated by nanoformulations were beyond the scope of this work. Moreover, while the cuprizone model is an established and widely used model for demyelination, it does not completely replicate the complex pathophysiology of human multiple sclerosis, particularly with regard to immune cell infiltration and remyelination dynamics. Future studies could explore the effects of these nanoformulations in more complex models of chronic demyelination or experimental autoimmune encephalomyelitis, which more closely mimic the immune-mediated aspects of multiple sclerosis.

Despite these limitations, the findings of this study hold significant clinical implications. A major strength of this study is the comprehensive evaluation of both behavioral and molecular markers across demyelination and remyelination phases, providing a holistic view of therapeutic efficacy. The ability of curcumin and curcumin–piperine nanoformulations to support remyelination and improve motor function offers a potential therapeutic strategy for treating demyelinating disorders, particularly multiple sclerosis. The use of nanoformulations to enhance bioavailability and target delivery further improves their clinical relevance, providing an efficient method for delivering bioactive compounds to the central nervous system. Given the increasing recognition of oxidative stress and inflammation in the pathogenesis of neurodegenerative diseases, future research into the development of novel, effective neuroprotective therapies based on natural products like curcumin could offer valuable alternatives or adjuncts to current treatments.

## 4. Materials and Methods

### 4.1. Animals

Seventy-five Swiss albino male mice (SWR/J) weighing 21–25 g were acquired from the King Fahd Medical Research Center (KFMRC) animal housing unit at King Abdulaziz University in Jeddah, Saudi Arabia. A 12 h light/dark cycle, with the light cycle occurring between 7:00 am and 7:00 pm, was implemented to maintain the mice at a suitable room temperature of 23 ± 2 °C and humidity level of 65%. Food and water were freely available to each mouse. Animal studies were performed in compliance with the KFMRC Animal Unit Committee rules. The Biomedical Ethics Research Committee at King Abdulaziz University authorized the study protocol (approval No. ACUC-22-1-2), in compliance with the guidelines established by the Animal Care and Use Committee of the KFMRC. The research adhered to the “System of Ethics”. The study was authorized by Royal Decree No. M/59 dated August 24, 2010, and conformed with the “System of Ethics of Research on Living Creatures” rules created by King Abdulaziz City for Science and Technology.

### 4.2. Drug Preparations

CPZ (C9012-25G) was obtained from Sigma-Aldrich (Bangalore, India). As previously mentioned, mice were given 0.2% *w*/*w* CPZ combined with ground rodent chow for five weeks in order to cause acute demyelination [57,58]. By dissolving it in *Z. rhetsa* Seed Oil (ZRO), as previously published and defined, a nanoformulation of curcumin with and without piperine was created [29]. Briefly, these nanoformulations were created by adding varying ratios of surfactant, cosurfactant, and/or cosolvent to the ZRO. ZRO was formulated using a fixed amount of cosurfactant (I988) and Tween 85 to examine its effect on the formulation performance. The resulting liquid was homogenized and stored for further use. This is a self-nanoemulsifying drug delivery system (SNEDDS). The SNEDDS solubility values are 19.0 mg/g and 48.22 mg/g, respectively. The drug-free SNEDDS had a droplet size of 607.1 nm, whereas the drug-loaded SNEDDS had a droplet size of 700.86 nm with curcumin and piperine. The zeta potentials of curcumin–piperine-loaded SNEDDS (−36.35 mV) and drug-free SNEDDS (−36.9 mV) showed that the resulting emulsion had good physical stability. Following curcumin–piperine loading, the zeta potential value remained unchanged. Tween 85 and I988 were included as surfactant and co-surfactant, respectively, to stabilize the nanoemulsion and achieve optimal droplet size and drug dispersion in the SNEDDS system. While these excipients are pharmacologically inert, they play a critical formulation role and have no expected biological activity in vivo. Curcumin (10 mg/kg) was administered at a dosage determined by prior research [59]. A suitable quantity of piperine, blank, and curcumin with nanoformulation were further diluted in regular saline until 0.1 mL contained 3 mg/kg of piperine and 10 mg/kg of curcumin for each mouse weight. Every day from 11:00 a.m. to 1:00 p.m., 0.1 mL of the formulation was administered intraperitoneally to each mouse. Weekly measurements of mouse weights were used to modify the dosages.

### 4.3. GC-MS Characterization of Zanthoxylum rhetsa Seed Oil

A Perkin Elmer Model Clarus 600 T combined with a single quadrupole mass spectrometer was used for GC-MS analysis. The chromatographic column was an Elite 5 MS column (30 m × 0.25 mm × 0.25 µm film thickness), with high-purity helium as the gas carrier, at a flow rate of 1 mL/min. The injector temperature was 280 °C and it was equipped with a splitless injector at 20:1 and sample injection volume 1 microliter. The temperature was set initially to 40 °C (held for 1 min), then was increased to 150 °C at the ramp temperature of 10 °C per minute (held for 1 min), then increased further to 300 °C at the ramp temperature of 10 °C per minute for 1 min. The MS ion source temperature was 220 °C and inlet line temperature was set to 240 °C. The scan range was set at 40 to 600 mass ranges at 70 eV electron energy having multiplier voltage at 270 and the solvent delay of 4 min. Finally, unknown compounds were identified by comparing the spectra with that of the NIST 2005 (National Institute of Standard and Technology library) and Wiley 2006 library. The total time required for analyzing a single sample was 29 min.

### 4.4. Experimental Design

The study duration was seven weeks, comprising five weeks of demyelination followed by two weeks of remyelination. Fifteen mice from each group were randomly allocated to five primary groups. (1) The control group received standard chow and 0.1 mL of saline intraperitoneally (i.p.) for seven weeks. (2) The cuprizone (CPZ) group was administered CPZ-mixed chow and 0.1 mL of normal saline (i.p.) for five weeks. (3) This group also received a 0.1 mL ZRO-based blank formulation administered (i.p.) along with CPZ-mixed chow daily. (4) The fourth group was administered CPZ-mixed chow and a ZRO-based formulation of curcumin (0.1 mL i.p.) for five weeks. (5) The fifth group was administered CPZ-mixed chow and a ZRO-based nanoformulation of curcumin with piperine for five weeks. Cuprizone feeding was discontinued in all groups at the conclusion of the demyelination stage (week 5); however, treatment with various nanoformulations continued until the end of week 7. Group designations were as follows: (1) control group, (2) Cuprizone-containing CPZ group, (3) Blank formulation or vehicle BFZ group, (4) Curcumin alone CFZ group, and (5) Curcumin with piperine PFZ group.

### 4.5. Sample Collection

At the conclusion of the fifth week, a random selection of half of the mice in each group was subjected to isoflurane anesthesia and subsequently decapitated to terminate their lives. The entire brain of each mouse was then extracted through dissection. Further dissection was performed on the obtained brains to isolate specific brain regions, including the hippocampus, frontal cortex, cerebral cortex, brain stem, and cerebellum. These brain sections were immersed in RNA-later solution and preserved at −80 °C for future analysis. The remaining mice underwent the same procedure at the end of week 7.

### 4.6. Grip Strength Test

Neuromuscular function in rodents was evaluated using the grip strength test. In this experiment, the maximal peak force a rat can generate when pulled from a specially designed grid by the experimenter is measured. The forelimb strength (g force) was determined using a grip strength meter (Columbus Instruments, Columbus, OH, USA). Mice were briefly restrained by their tails and allowed to grasp the grids with their forelimbs. Once the mice had established a firm grip, they were pulled away until the grip was released, at which point the grip-force value was recorded. The experiment was conducted according to the protocol of Takeshita et al. In brief, each mouse underwent three trials of the test, after which the mean results (g) were documented and normalized to body weight for each mouse [60].

### 4.7. Open Field Test

We employed an established technique commonly utilized for assessing motor function in rats to conduct the open field test on a weekly basis, with the aim of evaluating the effects of cuprizone and melatonin on motor behavior [61]. At the commencement of the test, each mouse was placed in an open-field arena measuring 45 cm by 45 cm square, and it was allowed to ambulate freely for three minutes. A camera mounted above the enclosure tracked and recorded the mice’s movements. Both velocity (cm/s) and total distance traveled (TDM, in cm) were quantified using the EthoVision tracking system.

### 4.8. Rotarod Test

The Rotarod test, a behavioral assessment tool, was initially introduced by Dunham and colleagues in 1957. Its purpose is to evaluate several motor-related parameters in rats, including balance, coordination, and fatigue [62]. Utilizing a Rotarod apparatus (BR1001, B.S Technolab Inc., Seoul, Republic of Korea), the effects of CPZ and interventions on motor coordination and balance during demyelination and remyelination, respectively, were assessed in this study. Specifically, the equipment was deactivated and the time at fall was recorded if a mouse fell off the rotating rod. Each mouse was positioned on the rod at 4 rpm, which was then steadily accelerated to 40 rpm over a period of 300 s. On each test day, a mouse underwent three trials, separated by a 40–60 min rest interval, and the mean latency to fall was recorded [63]. The “latency to fall” is employed as a quantitative endpoint to evaluate motor function.

### 4.9. Histological Examination

At the conclusion of week five, or the demyelination stage, whole brains were extracted and preserved in 10% formalin for 48 h. Whole brain specimens were subsequently fixed in paraffin wax, and sections were cut at a thickness of 4 µ using a LEICA-RM2255 (Leica Microsystems, Wetzlar, Germany) before being mounted onto glass slides for staining. Luxol Fast Blue (LFB) eosin and hematoxylin (H&E) staining was employed to examine the pathohistological alterations induced by cuprizone toxicity (demyelination). At the end of week seven, the identical procedure was repeated (remyelination stage). Histological images of the hippocampal regions were captured using an Olympus BX53 microscope and an Olympus DP73 camera at various magnifications. The images were subsequently analyzed using Olympus CellSens Entry software Version 4.2 (Olympus Corporation, Tokyo, Japan).

### 4.10. Biochemical Analysis

Tissues were homogenized in RIPA lysis buffer (R-0278; Sigma) containing PMSF at the prescribed doses, Halt^TM^ phosphatase inhibitor (Thermo-Fisher Scientific, Waltham, MA, USA), and protease inhibitor cocktail (cOmplete^TM^, Roche, Indianapolis, IN, USA) for use in various biochemical assays. After centrifuging the homogenate at 15,000× *g* for 20 min, the supernatant was separated and stored for further biochemical examination. With the use of an assay kit obtained from SolarBio (Beijing, China), the SOD and CAT levels were determined. ELISA kits from Sunlong Biotech Co., Ltd. (Hangzhou, China) were used to measure the levels of CD4, CD8, MBP, GSH, GPX, BDNF, CREB, TNFα, and IL-1β in accordance with the manufacturer’s instructions.

### 4.11. RNA Extraction and cDNA Synthesis

Total RNA was extracted from the frontal cortex using a Total RNA extraction kit (R1200) from Solarbio, China. In short, after being taken out of the RNA later solution at −80 °C, the brains were let to thaw at ambient temperature. In tubes, 50–60 mg of homogenized frontal cortex tissue was used for RNA extraction, following the manufacturer’s instructions. A NanoDrop spectrophotometer was used to measure the concentration and purity of the RNA, and the samples were kept at −80 °C to facilitate cDNA synthesis. Following the manufacturer’s instructions, the RNAs were reverse transcribed to cDNA using a Maxima First Strand cDNA Synthesis Kit K1641 (Thermo-Fisher Scientific).

### 4.12. Gene Expression Analysis

This study examined the amounts of mouse BDNF, CREB, TNFα, and IL-1β mRNA expression. Table 6 lists the primer sequences and amplicon sizes for the gene-specific primers that were created using the NCBI primer design tool. In a nutshell, 300 nM forward and reverse primers were combined with PowerUp SYBR Green PCR Master Mix (Applied Biosystems, Waltham, MA, USA) and 10 ng of the generated cDNA. The PCR process was initiated according to the kit’s instructions: (1) UDG activation for two minutes at 50 °C (one cycle); (2) Dual-LockTM DNA polymerase activation for two minutes at 95 °C (one cycle); and (3) forty cycles of denaturation for fifteen seconds at 95 °C followed by annealing/extension for one minute at 60 °C (if primer Tm ≥ 60 °C). The annealing temperature (52–59 °C) was established for each primer set including primers with a Tm less than 60 °C, and the temperature was eventually raised to 72 °C. For the purposes of this investigation, the housekeeping gene was beta-2-microglobulin (B2M). The StepOne device (Applied Biosystems) was used to conduct the research. Utilizing the delta-delta Ct technique, the relative fold change in gene expression was determined.

### 4.13. Statistical Analysis

The changes and comparisons between different parameters at the end of demyelination and remyelination stage were statistically analyzed using one-way ANOVA followed by Tukey’s post hoc test was used for all intergroup comparisons. Alterations in the results were considered significant when the *p* value was ≤0.05. Most of the statistical analyses were performed using Microsoft Excel and Social Science Statistics freely available at https://www.socscistatistics.com/ accessed on 20 February 2025.

## 5. Conclusions

In this study, we demonstrated that cuprizone-induced demyelination leads to significant impairments in motor function, as evidenced by reductions in total distance moved, velocity, grip strength, and motor coordination. Treatment with curcumin and curcumin–piperine nanoformulations exhibited promising protective effects during the active demyelination phase and facilitated recovery after cuprizone cessation. These nanoformulations showed potential in modulating oxidative stress, reducing inflammation, and promoting remyelination, thereby improving motor performance. The results suggest that curcumin and curcumin–piperine, especially in nanoformulation form, may offer a novel therapeutic approach for treating demyelinating diseases such as multiple sclerosis. Further studies focusing on the molecular mechanisms underlying these effects, as well as the long-term impact of nanoformulation therapy, are warranted to fully explore their clinical potential.

## Figures and Tables

**Table 1:** Major peaks identified during GC-MS analysis of *Zanthoxylum rhetsa* seed oil.

S. No.	Chemical Constituents	RT (Minutes)	Area %
1.	HEXANAL	4.24	1.250
2.	HEXANOIC ACID	6.92	0.310
3.	2,4-HEPTADIENAL	7.36	0.790
4.	1-ETHYL-CYCLOHEXENE	7.60	1.170
5.	TETRAHYDRO-5-METHYL-2-FURANMETHANOL	8.33	0.480
6.	4-METHYL-PHENOL	8.55	0.280
7.	NONANAL	9.07	0.230
8.	OCTANOIC ACID	9.96	0.300
9.	1-DODECANOL	11.40	0.420
10.	(3Z,6Z)-DODECA-3,6-DIEN-1-OL	11.80	0.220
11.	2,4-DECADIENAL	12.20	4.460
12.	DIHEXYLSULFIDE	12.65	0.250
13.	TRANS-2-UNDECENAL	12.89	0.420
14.	OXACYCLOTETRADECANE-2,11-DIONE	14.22	0.770
15.	HEXADECANOIC ACID	20.55	28.420
16.	OCTADEC-9-ENOIC ACID	22.30	59.220
17.	METHYL ALPHA-KETOPALMITATE	25.00	0.410
18.	1-TETRADECENE	26.69	0.600

RT stands for Retention Time.

**Table 2:** Effect of CPZ, BFZ, CFZ and PFZ on antioxidant levels in frontal cortex.

Group	Antioxidant Activity							
	CAT U/mg Protein		SOD U/mg Protein		GSH ng/mg Protein		GPX ng/mg Protein	
	Week 5	Week 7	Week 5	Week 7	Week 5	Week 7	Week 5	Week 7
CNT	7.85 ± 0.55	8.22 ± 0.42	18.97 ± 1.15	28.38 ± 2.15	111.65 ± 4.05	120.33 ± 13.74	4.34 ± 0.91	3.61 ± 0.02
CPZ	5.59 ± 0.21	7.92 ± 0.08	12.34 ± 0.56	14.78 ± 1.99	57.28 ± 3.13	149.57 ± 16.87	2.24 ± 0.08	2.42 ± 0.07
BFZ	3.61 ± 0.38	6.44 ± 0.19	9.00 ± 0.94	19.25 ± 1.87	67.74 ± 3.14	132.03 ± 11.35	1.08 ± 0.13	4.22 ± 0.04
CFZ	6.74 ± 0.14	6.94 ± 0.14	11.25 ± 0.38	17.28 ± 0.92	74.44 ± 10.61	147.32 ± 14.9	1.04 ± 0.02	4.39 ± 0.03
PFZ	8.51 ± 1.16	10.42 ± 0.25	18.18 ± 0.98	34.20 ± 0.51	126.25 ± 15.7	138.01 ± 16.31	3.46 ± 0.34	3.35 ± 0.62
*p* value	* CNT × CPZ; BFZ × CFZ ** CNT × BFZ; CFZ × PFZ *** CPZ × PFZ **** BFZ × PFZ ns = CNT × BFZ, CFZ; CPZ × BFZ, CFZ	* CPZ × BFZ * CNT × BFZ, CFZ; CPZ × CFZ; BFZ × PFZ; CFZ × PFZ *** CNT × PFZ; CPZ × PFZ ns = CNT × CPZ; BFZ × CFZ	** CNT × CPZ; CPZ × PFZ; CFZ × PFZ **** CNT × BFZ *** CNT × CFZ; BFZ × PFZ ns = CNT × PFZ; CPZ × BFZ, CFZ; BFZ × CFZ	* CNT × PFZ; CPZ × BFZ, CFZ *** CNT × CPZ, BFZ; BFZ × CFZ, PFZ; CFZ × PFZ **** CNT × CFZ; CPZ × PFZ	* CNT × BFZ; CFZ × PFZ ** BFZ × PFZ **** CNT × CPZ; CPZ × BFZ, CFZ, PFZ ns = CNT × CFZ, PFZ; BFZ × CFZ	** CFZ × PFZ; BFZ × CFZ, PFZ; CPZ × BFZ; CNT × CFZ, PFZ *** CNT × CPZ; CPZ × PFZ ns = CNT × BFZ; CPZ × CFZ	* CNT × CPZ; BFZ × PFZ; CFZ × PFZ ** CNT × BFZ, CFZ ns = CNT × PFZ; CPZ × BFZ, CFZ, PFZ; BFZ × CFZ	** CPZ × BFZ, CFZ ns = all other interactions were non-significant

Data are shown as mean ± standard error of the mean; CAT, catalase; SOD, superoxide dismutase; GPX, glutathione peroxidase; GSH, glutathione. One-way ANOVA with Tukey’s multiple comparison test was performed to compare the variance between groups. * *p* ≤ 0.05, ** *p* ≤ 0.01, *** *p* ≤ 0.001, **** *p* ≤ 0.0001, ns = nonsignificant.

**Table 3:** Levels of CD4 and CD8 in frontal cortex at the end of Week 5 and Week 7.

Group	CD4 (pg/mg Protein)			CD8 (ng/mg Protein)		
	Week 5	Week 7	% Change	Week 5	Week 7	% Change
CNT	2725.25 ± 213.66	2585.69 ± 231.56	5.12 ↓	3.83 ± 0.46	5.18 ± 0.42	35.24 ↑
CPZ	18,466.57 ± 162.34	17,071.16 ± 138.48	7.56 ↓	11.93 ± 1.41	5.86 ± 0.74	50.88 ↓
BFZ	4557.21 ± 394.18	4497.18 ± 406.74	1.32 ↓	5.34 ± 0.68	3.65 ± 0.26	31.65 ↓
CFZ	3540.72 ± 276.44	1795.39 ± 128.62	49.29 ↓	3.35 ± 0.24	3.04 ± 0.22	6.25 ↓
PFZ	3302.33 ± 336.12	1298.19 ± 96.78	60.68 ↓	2.87 ± 0.22	2.26 ± 0.18	21.25 ↓
*p* value	**** CNT × CPZ **** CPZ × BFZ, CFZ, PFZ ** BFZ × CNT * CNT × CFZ, PFZ NS = CFZ × PFZ	**** CNT × CPZ **** CPZ × BFZ, CFZ, PFZ *** BFZ × CNT ** CNT × BFZ ** PFZ × CNT * CFZ × CNT		**** CNT × CPZ **** CPZ × BFZ, CFZ, PFZ ** BFZ × CNT * CFZ × PFZ NS = CNT × CFZ	NS = CNT × CPZ *** CNT × PFZ ** CNT × BFZ, CFZ * PFZ × CFZ NS = BFZ × CFZ	

One-way ANOVA with Tukey’s multiple comparison test was performed to compare the variance between groups. * *p* ≤ 0.05, ** *p* ≤ 0.01, *** *p* ≤ 0.001, **** *p* ≤ 0.0001; NS = nonsignificant. ↑ & ↓ represent increase and decrease in amount of protein respectively.

**Table 4:** BDNF, CREB, TNFα, and IL-1β protein levels in the frontal cortex at the end of demyelination stage.

Biomarker	Group	Concentration (pg/mg Protein)	Percent (%) Change in Relation to		*p*-Value
			CNT	CPZ	
BDNF	CNT	542.16 ± 30.42	0.00	35.07 ↑	* CPZ × CFZ; * BFZ × CFZ; * CFZ × PFZ; ** Control × CPZ, BFZ, PFZ; ** CPZ × BFZ, PFZ; NS = CNT × CFZ; BFZ × PFZ
	CPZ	401.39 ± 36.74	25.96 ↓	0.00	
	BFZ	801.24 ± 74.10	47.79 ↑	99.62 ↑	
	CFZ	596.41 ± 23.64	10.01 ↑	48.59 ↑	
	PFZ	853.84 ± 43.15	57.49 ↑	112.72 ↑	
CREB	CNT	1859.19 ± 83.67	0.00	44.66 ↑	** CNT × CPZ; ** CPZ × BFZ; *** CPZ × CFZ, PFZ; *** CNT × CFZ, PFZ; **** BFZ × CFZ, PFZ; NS = CNT × BFZ
	CPZ	1285.22 ± 64.28	30.87 ↓	0.00	
	BFZ	1803.64 ± 92.37	2.99 ↓	40.34 ↑	
	CFZ	473.79 ± 27.61	74.52 ↓	63.14 ↓	
	PFZ	402.06 ± 17.55	78.37 ↓	68.72 ↓	
TNFα	CNT	373.03 ± 13.67	0.00	15.71 ↓	* CNT × CFZ; * CPZ × BFZ, CFZ; ** CNT × CPZ; **** CNT × PFZ; CPZ × PFZ; BFZ × PFZ; CFZ × PFZ; NS = CNT × BFZ; BFZ × PFZ
	CPZ	442.55 ± 29.47	18.64 ↑	0.00	
	BFZ	347.46 ± 16.52	6.85 ↓	21.49 ↓	
	CFZ	312.41 ± 23.25	16.25 ↓	29.41 ↓	
	PFZ	636.5 ± 56.32	70.63 ↑	43.83 ↑	
IL-1β	CNT	66.79 ± 4.86	0.00	24.01 ↓	* CNT × CPZ, CFZ; * BFZ × CFZ, PFZ; ** CNT × PFZ; *** CPZ × CFZ, PFZ; NS = CNT × BFZ; CFZ × PFZ
	CPZ	87.89 ± 5.94	31.59 ↑	0.00	
	BFZ	63.69 ± 3.97	4.64 ↓	27.53 ↓	
	CFZ	46.59 ± 3.16	30.24 ↓	46.99 ↓	
	PFZ	40.89 ± 2.95	38.78 ↓	53.48 ↓	

One-way ANOVA with Tukey’s multiple comparison test was performed to compare the variance between groups. * *p* ≤ 0.05, ** *p* ≤ 0.01, *** *p* ≤ 0.001, **** *p* ≤ 0.0001, NS = nonsignificant. ↑ & ↓ represent increase and decrease in amount of protein respectively.

**Table 5:** BDNF, CREB, TNFα, and IL-1β protein levels in frontal cortex at the end of remyelination stage.

Biomarker	Group	Concentration (pg/mg Protein)	Percent (%) Change in Relation to		*p*-Value
			CNT	CPZ	
BDNF	CNT	1047.09 ± 77.92	0.00	114.29 ↑	* CPZ × BFZ; * BFZ × CFZ; * CFZ × PFZ ** CNT × BFZ, PFZ *** CNT × CPZ, CFZ NS = BFZ × PFZ
	CPZ	488.64 ± 36.19	53.33 ↓	0.00	
	BFZ	762.35 ± 74.62	27.19 ↓	56.01 ↑	
	CFZ	554.95 ± 35.68	47.00 ↓	13.57 ↑	
	PFZ	733.90 ± 61.29	29.91 ↓	50.19 ↑	
**CREB**	CNT	917.7 ± 38.53	0.00	107.72 ↑	*** CPZ × BFZ; * BFZ × CFZ, PFZ **** CNT × CPZ, CFZ, PFZ NS = CNT × BFZ; CPZ × CFZ, PFZ
	CPZ	441.8 ± 23.61	51.86 ↓	0.00	
	BFZ	839.47 ± 44.39	8.52 ↓	90.01 ↑	
	CFZ	364.91 ± 19.46	60.24 ↓	17.40 ↓	
	PFZ	470.51 ± 15.94	48.73 ↓	6.50 ↑	
**TNFα**	CNT	529.45 ± 62.31	0.00	1.22 ↓	* BFZ × PFZ *** CNT × BFZ; *** CPZ × BFZ **** CNT × CFZ, PFZ; *** CPZ × CFZ, PFZ NS = CNT × CPZ; BFZ × CFZ; CFZ × PFZ
	CPZ	536.0 ± 36.24	1.24 ↑	0.00	
	BFZ	338.24 ± 26.34	36.11 ↓	36.90 ↓	
	CFZ	270.3 ± 23.31	48.95 ↓	49.57	
	PFZ	227.47 ± 30.85	57.04 ↓	57.56 ↓	
**IL-1β**	CNT	51.76 ± 3.14	0.00	109.81 ↑	** CNT × CFZ; ** BFZ × CFZ *** CNT × CPZ; CFZ × PFZ; **** CNT × BFZ, PFZ; **** CPZ × BFZ, CFZ, PFZ; NS = BFZ × PFZ
	CPZ	24.67 ± 2.54	52.34 ↓	0.00	
	BFZ	98.95 ± 6.78	91.17 ↑	301.09 ↑	
	CFZ	74.37 ± 4.32	43.68 ↑	201.46 ↑	
	PFZ	102.77 ± 6.71	98.55 ↑	316.58 ↑	

One-way ANOVA with Tukey’s multiple comparison test was performed to compare the variance between groups. * *p* ≤ 0.05, ** *p* ≤ 0.01, *** *p* ≤ 0.001, **** *p* ≤ 0.0001, NS = nonsignificant. ↑ & ↓ represent increase and decrease in amount of protein respectively.

**Table 6:** PCR primers used in this study.

Gene	Primer Orientation	Sequence	Amplicon Size (bp)
*BDNF*	Forward	AAGGGCCAGGTCTGTTAATCG	70
	Reverse	ATGGCTCTATGAAACTGTTGTGGT	
*TNFα*	Forward	GGTCCCCAAAGGGATGAGAAGT	124
	Reverse	TTGCTACGACGTGGGCTAC	
*CREB*	Forward	ACCCACGAGCACCATTCGC	120
	Reverse	TGCCTCCCTGTTCTTCATTAGA	
*IL-1β*	Forward	CCCCAAAAGATGAAGGGCTGC	108
	Reverse	TGCCTGCCTGAAGCTCTTGT	

## Data Availability

Data can be obtained from the corresponding author upon request.
